# Terrestriality and bacterial transfer: a comparative study of gut microbiomes in sympatric Malagasy mammals

**DOI:** 10.1038/s41396-018-0251-5

**Published:** 2018-08-14

**Authors:** Amanda C. Perofsky, Rebecca J. Lewis, Lauren Ancel Meyers

**Affiliations:** 1 Department of Integrative Biology, The University of Texas at Austin, Austin, TX USA; 2 Department of Anthropology, The University of Texas at Austin, Austin, TX USA; 3 Santa Fe Institute, Santa Fe, NM USA

**Keywords:** Microbiome, Community ecology, Phylogenetics

## Abstract

The gut microbiomes of mammals appear to mirror their hosts’ phylogeny, suggesting host-driven microbial community assembly. Yet, much of this evidence stems from comparative studies of distinct wild or captive populations that lack data for disentangling the relative influences of shared phylogeny and environment. Here, we present phylogenetic and multivariate analyses of gut microbiomes from six sympatric (i.e., co-occurring) mammal species inhabiting a 1-km^2^ area in western Madagascar—three lemur and three non-primate species—that consider genetic, dietary, and ecological predictors of microbiome functionality and composition. Host evolutionary history, indeed, appears to shape gut microbial patterns among both closely and distantly related species. However, we also find that diet—reliance on leaves versus fruit—is the best predictor of microbiome similarity among closely related lemur species, and that host substrate use—ground versus tree—constrains horizontal transmission via incidental contact with feces, with arboreal species harboring far more distinct communities than those of their terrestrial and semi-terrestrial counterparts.

## Introduction

The gastrointestinal tracts of mammals are complex ecosystems harboring large and diverse populations of bacteria that are essential for digestion, development, metabolism, behavior, immune function, and protection from pathogens [1–6]. These microbial communities are potentially shaped by diverse host factors—heritable (e.g., genetics, evolutionary history), environmental (e.g., geography, diet), as well as behavioral (i.e., social contact patterns) [7]. Hypothetically, if microbial composition is primarily host-driven, with gut bacteria colonizing hosts strictly via maternal inheritance (i.e., vertical transmission) or intraspecific transmission, then the ecological relatedness among gut microbial communities should mirror their hosts’ phylogeny [8, 9]. Conversely, if microbial community assembly is stochastic, with bacteria horizontally transmitted among distantly related hosts via shared food sources or habitat, then animal species with overlapping diets and environmental exposures should harbor similar microbiomes, regardless of their evolutionary divergence [9]. However, dietary and habitat preferences can obscure the phylogenetic signal within host-associated microbial communities, thus confounding attempts to estimate the relative influences of vertical, horizontal, and environmental transmission on mammalian gut microbiome composition [9].

Several studies have estimated the relative influences of host phylogeny, genetics, diet, and environment on both interspecific and intraspecific microbiome diversity in mammals. Several studies of zoo animals reared on artificial diets have claimed that diet and shared environment have greater impacts on gut microbiome composition than endogenous factors [10–14]. Other studies have focused on closely related mammal species and generally found that host species and their gut microbes exhibit concordant relationships [8, 15–18], with the gut microbial communities of different host species forming distinct clusters [18–20]. Within single host species, some studies of humans and other primates have suggested that social contact patterns are the primary predictor of gut microbiome composition [21–25], whereas others have found that host genetics exerts a strong influence [26, 27].

Most of these comparative microbiome surveys have sampled geographically and ecologically distinct captive or wild populations (but see [14, 27, 28]). However, captivity alters microbiome composition in mammals [12, 29–31], and, when animals are compared across geographic regions, host phylogenetic differences may be confounded by differences in local microbial taxa [15, 32]. Thus, many of these prior studies did not have sufficient data for resolving the relative influences of various heritable and environmental factors.

Madagascar is home to a unique and threatened constellation of mammalian fauna, with high levels of endemism and broad species diversity across remarkably few taxonomic groups [33, 34]. This phenomena has been attributed to Madagascar’s long isolation from other continents, which predates the evolution of most recent mammals, and rare “sweepstake” migration events over the past 65 million years [35, 36]. All non-flying mammals of Madagascar belong to either one of four endemic orders—lemurs, tenrecs, carnivorans, or rodents—or are recent human introductions—African bush pigs [37], cattle, cats, or dogs [38]. Recent studies have shown that the gut microbiomes of wild lemurs reflect social group membership and seasonal changes in diet [24, 25, 39, 40], but none yet have considered the microbiomes of co-occurring mammalian species.

Here, we present an analysis of microbiome diversity across six sympatric (i.e., geographically co-occurring) mammalian populations inhabiting a 1-km^2^ area in western Madagascar. The focal species include both folivorous and frugivorous lemurs (Verreaux’s sifaka, *Propithecus verreauxi*; red-tailed sportive lemur, *Lepilemur ruficaudatus*; red-fronted brown lemur, *Eulemur rufifrons*), a viverrid that is the largest extant carnivore in Madagascar (fossa, *Cryptoprocta ferox*), and two human-introduced artiodactyls (even-toed ungulates)—one wild (African bush pig, *Potamochoerus larvatus*) and the other domesticated (zebu cattle, *Bos t. indicus*). We utilize multivariate modeling and phylogenetic approaches to disentangle the relative contributions of host environment, diet, and evolutionary history on gut microbial community structure. We estimate microbiome diversity, composition, and functional potential using 16S rRNA sequences from 61 field-collected samples as well as published surveys and show that host phylogeny predicts compositional diversity on a broad scale, while host diet appears to exert a greater influence among recently diverged primate hosts. Furthermore, we find evidence of greater horizontal transmission between distantly related *terrestrial* (i.e., ground-dwelling) mammals, relative to closely related *arboreal* (i.e., tree-dwelling) species, thus suggesting that substrate preference (i.e., the primary surface upon which animals locomote) also shapes microbiome composition.

## Materials and methods

### Fecal sample collection

**Fig. 1:**
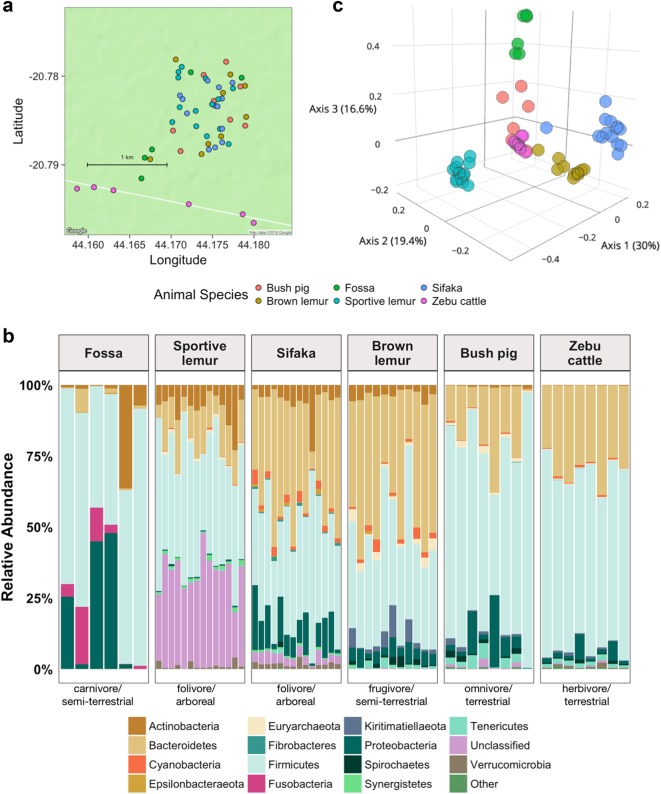
Sympatric mammal populations harbor distinct gut microbiotas. **a** Fecal sample collection sites at Ankoatsifaka Research Station in Kirindy Mitea UNESCO Biosphere in western Madagascar. **b** The relative abundances of the fourteen most abundant bacterial phyla in six Malagasy mammal species (fossa,* C. ferox*; red-tailed sportive lemur, *L. ruficaudatus*; Verreaux’s sifaka, *P. verreauxi*; red-fronted brown lemur, *E. rufifrons*; bushpig, *P. larvatus*; zebu cattle, *B. t. indicus*). The “Other” category represents low abundance (<2%) phyla. **c **Three-dimensional principal coordinates plot of normalized weighted Unifrac distances showing ecological distances among 61 microbiome samples from six mammal species

We collected a total of 61 fresh fecal samples from wild populations of Verreaux’s sifaka (*Propithecus verreauxi*), red-tailed sportive lemurs (*Lepilemur ruficaudatus*), red-fronted brown lemurs (*Eulemur rufifrons*), fossa (*Cryptoprocta ferox*), and African bush pigs (*Potamochoerus larvatus*) within a 1-km^2^ area at Ankoatsifaka Research Station (20°47.69′S, 44°9.88′E; Fig. 1a and S1) in Kirindy Mitea UNESCO Biosphere Reserve in western Madagascar (Table S1). We also collected zebu cattle (*Bos t. indicus*) samples along a 20 km stretch of dirt road that traverses Kirindy Mitea (Table S1). Detailed fecal sample collection methods are provided in the supplementary information. All samples were collected during a two-month span in the dry season (10 June 2012–1 August 2012) and preserved in RNAlater® (ThermoFisher, Waltham, MA, USA) at ambient temperature until their arrival at the University of Texas at Austin in August 2012. We subsequently stored samples at −80 °C until further processing.

### Host phylogeny and life history data

We estimated phylogenetic distances among mammal species using mitochondrial cytochrome b (cyt-b) gene sequences (methods in supplementary information). Phylogenetic relationships among the distantly related species mirrored evolutionary times estimated from a time-calibrated ultrametric phylogenetic tree reconstructed with 5020 species [41], and those within the Strepsirrhine clade reflected divergence times estimated from a ultrametric Primate phylogenetic tree [42].

We broadly categorized zebu cattle as herbivores (i.e., feeding on plant material), sifaka and sportive lemurs as flexible folivores (i.e., primarily feeding on plant photosynthetic material), brown lemurs as flexible frugivores (i.e., primarily feeding on fruit and seeds), bush pigs as omnivores (i.e., feeding on both plant and animal material), and fossa as carnivores (i.e., primarily consuming animal matter) (Table 1). We also used primary literature to categorize each mammal species by substrate use—“terrestrial”, “arboreal”, or “semi-terrestrial”—and gut physiology—“hindgut-fermenter”, “foregut-fermenter”, or “simple-gut” (Table 1). To quantify between-species differences in dietary intake, we used direct observations of feeding behavior for Verreaux’s sifaka at our research site and, for the remaining five species for which we did not have direct observation data, primary literature specific to dry deciduous forests in western Madagascar (Table 1). Our sifaka feeding data [24] were comparable to those reported in studies conducted for a different study population at Kirindy Forest Reserve [43, 44]. We computed a dietary distance matrix for the six mammal species based on the proportions of dietary items consumed by each species (methods in supplementary information).

**Table 1:** Mammal populations sampled in Kirindy Mitea Biosphere, Madagascar

Study species	Scientific name	Diet category	Gut physiology	Substrate use	Home range (km^2^)	Supplemental references
Zebu cattle	*Bos t. indicus*	Herbivore	Foregut fermenter	Terrestrial	NA	1, 2
African bush pig	*Potamochoerus larvatus*	Omnivore	Simple gut	Terrestrial	7.2	3–5
Fossa	*Cryptoprocta ferox*	Carnivore	Simple gut	Semi-terrestrial	9.2	1, 6–8
Verreaux’s sifaka	*Propithecus verreauxi*	Flexible folivore	Hindgut fermenter	Arboreal	0.15	9–14
Red-tailed sportive lemur	*Lepilemur ruficaudatus*	Flexible folivore	Hindgut fermenter	Arboreal	<0.01	15–18
Red-fronted brown lemur	*Eulemur rufifrons*	Flexible frugivore	Simple gut	Semi-terrestrial	0.21	9, 19, 20

### Fecal sample DNA extraction and 16S amplicon sequencing

We extracted DNA from 100 to 200 μg fecal pellets using a phenol chloroform bead-beating procedure [45]. For each set of DNA extractions, an empty tube was exposed to laboratory air and processed as a negative control. We quantified DNA samples with Picogreen® reagent (ThermoFisher) on a Qubit® fluorometer (ThermoFisher) and PCR amplified the V4 hypervariable region of the bacterial 16S ribosomal RNA gene using primers 515F and 806R [46]. The resulting barcoded amplicons were pooled and paired-end sequenced in 2 × 151 mode using the Illumina MiSeq platform at Argonne National Laboratory (Lemont, IL).

### Sequence processing and taxonomic assignments

To assess strain-level variation in microbial communities, we demultiplexed raw Illumina sequence reads using QIIME 1 [47] and then processed these reads using the DADA2 pipeline [48], which implements a parametric model of Illumina amplicon errors to reconstruct exact biological sequences (i.e., amplicon sequence variants, ASVs). We assigned taxonomic classifications to ASVs with a naïve Bayesian classifier based on their best match in the Silva reference database v132. After initial quality filtering and chimera removal, our dataset contained 4,119,513 processed paired-end sequence reads, averaging 67,533 reads per fecal sample. We removed singletons, ASVs designated as “unclassified” or ‘Eukaryota’ at the kingdom level, and chloroplast and mitochondrial ASVs. The resultant set of ASVs contained a total of 4,117,611 reads ($\overline x$ = 67,502 ± 13,535 s.d. reads per sample, range: 31,191–106,700) and 6146 unique ASVs ($\overline x$ = 321 ± 200 s.d. ASVs per sample, range: 62–874).

### Microbial diversity and composition among animal species

We conducted all statistical analyses using the statistical computing software R version 3.2.4 [49] and used the *ggplot2* [50] and *cowplot* [51] packages and Plotly [52] for visualization.

### Gut microbial richness

To test for differences in within-sample richness and evenness among microbiomes, we generated 100 ASV tables rarefied to 31,191 reads (the smallest library size in the dataset) for each individual sample. After rarefaction, individual samples contained 62–871 unique ASVs ($\overline x$ = 318 ± 198 s.d. ASVs per sample). We calculated mean rarefied richness (number of observed sequence variants) and Shannon’s diversity index for each sample using the rarefied ASV tables. We used Kruskal–Wallis tests adjusted for multiple comparisons to evaluate whether bacterial taxa and evenness per sample differed across animal species.

### Sample clustering

We assessed similarity in gut microbial communities using only sequence variants that were detected in at least two samples, totaling 3,846,102 reads ($\overline x$ = 63,051 ± 14,226 s.d. reads per sample, range: 30,328–94,387) and 2323 unique ASVs. To account for differences in sequencing depth among samples and heteroscedasticity in ASV counts, we estimated sample-specific normalization factors using the *DESeq2* package [53] and then rescaled the ASV counts. After normalization, samples contained 38–576 unique ASVs ($\overline x$ = 244 ± 132 s.d.). We conducted multivariate and community analyses using the *vegan* and *phyloseq* packages [54, 55]. We quantified inter-individual variation in gut microbial composition by calculating weighted Unifrac distances and Bray-Curtis dissimilarities between samples and performed principal coordinate analysis and complete linkage hierarchical clustering to visualize microbiome distances. We used permutational multivariate analysis of variance (PERMANOVA) to assess differences in composition according to host species and substrate preference (1000 permutations) and a linear mixed-effect model to assess pairwise predictors of microbial similarity (methods provided in supplementary information). We performed clustering of taxonomic profiles via partitioning of data around medoids (PAM) using the *cluster* and *fpc* packages [56].

### Random forest classification

We determined the degree to which individual microbiome samples can be classified into their respective host species by implementing a random forest classifier (RFC) supervised learning algorithm (*randomForest* package) [57]. We used all sequence variants in the normalized ASV dataset as predictors in the RFC model, with host species identity used as the category for RFC model distinguishability.

### Differentially abundant microbial taxa

We assessed differential abundance of bacterial phyla, families, and genera among host species using the nonparametric SAMseq approach (*samr* package) [58]. This method uses repeated permutations for assessment of the false discovery rate (FDR). We limited analyses to bacterial and archaeal phyla that occurred at least 50 times, classifiable families that occurred at least 100 times, and classifiable genera that occurred at least 100 times in the dataset. We performed SAMseq analyses separately for each taxonomic level using 1000 permutations and 100 re-samplings and considered differential abundance significant if the FDR-adjusted *P* value was < 0.05. ﻿We used indicator species analysis (*indicspecies* package) [59] to determine which microbial genera are shared among arboreal (i.e., tree-dwelling) versus terrestrial (i.e., ground-dwelling) mammal species (*P* < 0.05, indicator value > 0.5).

### Microbiome OTU-based analyses

We generated a closed-reference 97% operational taxonomic unit (OTU) dataset for use in a mammalian microbiome meta-analysis and to predict the metagenomic function of lemur microbiomes (methods in supplementary information).

### Microbiome composition and host phylogeny

We used a parsimony approach [8] to determine whether similarities in gut microbiome composition are congruent with the evolutionary relationships among mammalian host species (methods in supplementary information). As an alternative method for assessing phylogenetic congruence between gut microbial communities and their hosts, we used Mantel tests with 1000 permutations (*vegan* package) [55] between matrices of pairwise weighted Unifrac distances or Bray-Curtis dissimilarities and host phylogenetic divergence. We also performed partial Mantel tests to assess the correlation between host phylogeny and microbiome dissimilarity while controlling for geographic or dietary distance among samples.

## Results

### Microbiome composition

The gut microbial communities of the six mammalian host species encompassed two archaeal and 25 bacterial phyla, of which four (Firmicutes, Bacteroidetes, Proteobacteria, and Actinobacteria) were present in all samples and together constituted 51.2–99.7% of the reads identified in each individual (Fig. 1b). The microbial phyla, families, and genera associated with each animal species are detailed in the supplementary information (Table S2, Fig. S2). Bushpig and cattle microbiomes were enriched with Tenericutes, Planctomycetes, and Lentisphaerae whereas fossa microbiomes were deficient in Bacteroidetes and showed higher abundances of microbial taxa related to Firmicutes and Fusobacteria (SAMseq analysis, FDR-adjusted *P* *<* 0.0001; Table S2). Consistent with a recent microbiome study on folivorous primates [60], the microbiomes of sifaka and sportive lemurs included significantly higher proportions of unclassified reads at the phyla level (*P* *<* 0.0001; Table S2; sifaka: $\overline x$ = 0.03 ± 0.01 s.d. reads per sample; sportive lemurs: $\overline x$ = 0.33 ± 0.01), perhaps due to their remarkably different diet from that of humans. Sifaka and brown lemurs, the most closely related host species in our study, tended to share bacterial phylotypes belonging to Bacteroidetes and Proteobacteria (*P* *<* 0.0001; Table S2). Lastly, microbial lineages related to Spirochaetes that were comparatively abundant in brown lemurs were also enriched in cattle and bush pig samples (*P* *<* 0.0001; Table S2). Although we did not have data to show the presence of specific pathogens in our samples, several bacteria genera that include opportunistic pathogens, such as *Campylobacter, Clostridium*, and *Streptococcus*, were differentially abundant across mammal species (Fig. S2, Table S2).

Gut microbial diversity differed significantly across mammal species. The two artiodactyl species—cattle (herbivorous foregut fermenters) and bush pigs (omnivores with simple gut morphology)—harbored the most diverse microbiota (Kruskal–Wallis, FDR-adjusted *P* *<* 0.001; Fig. S3), whereas the three lemur species, two of which are hindgut fermenters [61, 62], exhibited moderate microbial diversity (Fig. S3). Fossa, the only carnivorous species in our study, had the fewest unique taxa and the lowest microbial species evenness (*P* *<* 0.001; Fig. S3). In accordance with Ley et al. [11], microbial diversity reflected dietary specialization, digestive physiology, and host taxonomy. However, our sample size of six host species was too small to test the statistical significance of these factors.

### Microbiome diversity across sympatric host species

Microbiome samples formed five discrete clusters (PAM clustering, Fig. S4) according to host species identity (PERMANOVA, weighted Unifrac metric: *R*^2^ = 0.74, *P* = 0.001, Bray-Curtis metric: *R*^2^ = 0.65, *P* = 0.001; RFC 98.4% cross-validation accuracy), with sifaka and sportive lemur microbiomes clustered independently and brown lemur microbiomes grouped more closely with those of fossa, cattle, and bush pigs (weighted Unifrac PCoA, Fig. 1c). Microbiome distances between animal species correlated significantly with substrate use (i.e., terrestriality versus arboreality), with terrestrial and semi-terrestrial species exhibiting similar microbial taxonomic structure and abundances (i.e., closer weighted Unifrac distances) despite having divergent diets and gut physiologies (PERMANOVA, weighted Unifrac: *R*^2^ = 0.2, *P* = 0.001, Bray-Curtis: *R*^2^ = 0.15, *P* *=* 0.001; GLMM, Table 2). In contrast, the microbiomes of the arboreal sifaka and sportive lemurs were highly divergent, despite their shared substrate preference and leaf-based diets (Fig. 1c). Indicator species analysis revealed that semi-terrestrial and terrestrial (i.e., ground-dwelling) mammals shared several anaerobic clostridial bacteria genera that are common inhabitants of human and animal gut microflora (*P* < 0.05, indicator value ≥ 0.5; Table S3). These indicator bacteria encompass both spore-forming and non-spore-forming species, suggesting that terrestriality may promote cross-species microbial exchange via either fecal-contaminated soil or direct contact with fecal material [63–65].

**Table 2:** Pairwise predictors of weighted Unifrac distance among six sympatric mammal species

Parameter	Estimate	95% CI	*z*-value	*P*-value	Interpretation
Intercept	−0.69	(−7.84, −0.6)	−14.47	**<2** **×** **10**^**−16**^	
Dietary distance	0.0009	(2.87 × 10^−5^, 0.002)	2.03	**0.043**	Host species with divergent diets have more dissimilar microbiota
Both terrestrial	−0.15	(−0.22, −0.08)	−4.05	**5.1** **×** **10**^**−5**^	Host species that both spend time on the ground have less dissimilar microbiota
Phylogenetic distance	2.31	(2.21, 2.41)	45.7	**<2** **×** **10**^**−16**^	Host species that are more evolutionary distant have more dissimilar microbiota
Geographic distance	−0.02	(−0.05, 0.006)	−1.56	0.12	No significant correlation

Average coefficient estimates, 95% confidence intervals, and *z*-values are shown for fixed-effect parameters. Bolded relationships are significant at *P* < 0.05. Baseline terrestriality (“no”) is not shown. Individual identities within each sample pair were included as random effects.

### Metagenomic functional analyses of lemur microbiomes

**Fig. 2:**
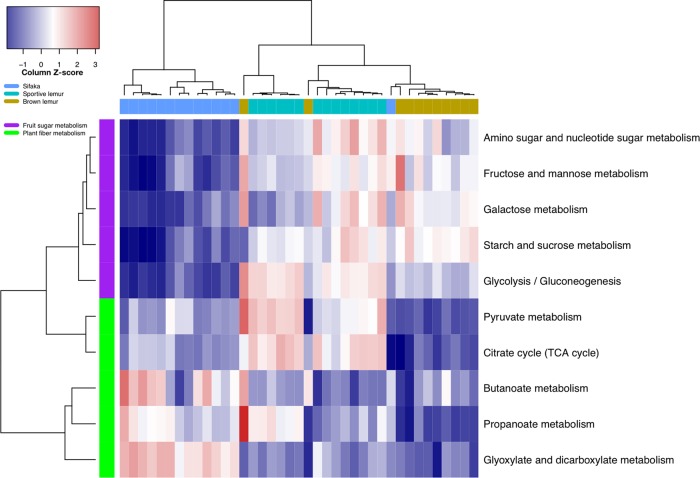
Heatmap of predicted carbohydrate metabolism in brown lemur, Verreaux’s sifaka, and sportive lemur microbiomes. The transformed relative abundances of the ten most abundant carbohydrate KEGG sub-pathways are shown, with the white color representing the relative abundance of pathways having the column average, blue tones indicating relative abundances less than the column average, and red tones indicating relative abundances greater than the column average. Brown lemur microbiomes are enriched for pathways associated with a frugivorous diet (purple rows), sifaka for those associated with a folivorous diet (green rows), and sportive lemurs for both frugivorous and folivorous pathways. Metagenomic functional profiles were inferred using PICRUSt. Metagenomic functional profiles and KEGG sub-pathways were hierarchically clustered using the UPGMA method

The gut microbiomes of the three lemur species had significantly distinct metabolic functionality (RFC 92.3% cross-validation accuracy, Fig. S5). Given the phylogenetic proximity of these species, we hypothesized that these functional differences may stem from diet intake rather than host evolutionary divergence. Sportive lemur microbiomes were enriched for both sugar metabolism and plant fiber degradation, consistent with a combined frugivorous and folivorous diet (Fig. 2). In contrast, brown lemur microbiomes were enriched for only sugar metabolism, with elevated levels of enzymes for metabolizing fructose, mannose, and sucrose, consistent with a fruit-based diet (Fig. 2). Sifaka microbiomes prioritized fiber degradation pathways, with enzymes for butanoate, propanoate, and glyoxylate metabolism, consistent with a leaf-based diet (Fig. 2). Pathways related to essential amino acid metabolism also distinguished the gut microbiotas of the lemur species and reflected disparities in protein content between fruit- and leaf-based diets (Fig. S6). Fruit pulp tends to be low in protein content, whereas leaves contain considerable amounts of rubisco, a protein involved in photosynthesis [66]. Hence, pathways for the biosynthesis of lysine and branched-chain amino acids (valine, leucine, isoleucine) were elevated in brown lemurs and sportive lemurs, whereas the catabolic reactions to break down these amino acids were enriched in sifaka (Fig. S6).

### Gut microbiome relationships mirror host phylogeny

**Fig. 3:**
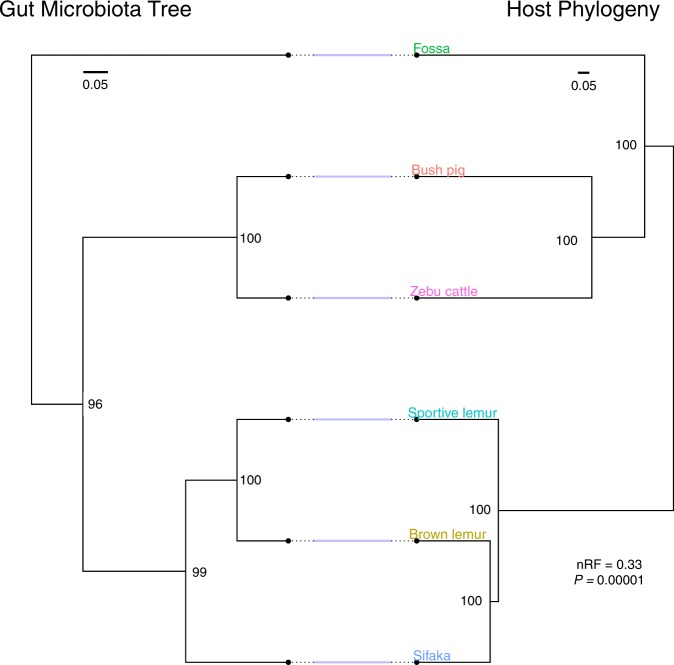
Comparison between gut microbiota tree and host phylogeny relationships. The gut microbiota tree reflects the same branching order as the host phylogeny for distantly related host species, but gut microbial similarity among sportive lemurs, brown lemurs, and sifaka does not match these hosts’ established evolutionary relationships. The maximum parsimony gut microbiota tree (left) is based on the abundances of 16S amplicon sequence variants (ASVs) in host species-aggregated microbiome samples. The maximum likelihood host species phylogeny (right) is based on mitochondrial cytochrome b (cyt-b) gene sequences. Topological congruence was quantified using the normalized Robinson-Foulds (nRF) metric, which calculates symmetry in rooted tree shape on a scale from 0.0 (complete congruence) to 1.0 (complete incongruence). The host phylogeny scale bar indicates nucleotide substitutions per site

We used phylogenetic methods to infer the pattern of hierarchical similarity among host species based on the frequencies and abundances of bacteria sequence variants in their representative gut microbiomes. The gut microbiota parsimony tree mirrored the host phylogeny for distantly related mammals, with the closely related sportive lemurs, brown lemurs, and sifaka clustering together separately from the other animal species (Fig. 3). However, the gut microbiota tree branching order did not reflect the fine-scale evolutionary relationships within the lemur clade (nRF = 0.33, *P* = 0.00001; Fig. 3). Specifically, the brown lemur and sifaka are more closely related animal species, yet the representative brown lemur microbiome was more similar to that of the sportive lemur. This finding is consistent with the metagenomic functional patterns and suggest that diet may confound host evolutionary history in structuring microbial communities among closely related sympatric primate species. When we analyzed individual microbiome samples rather than host species-aggregated microbiomes, lemur microbiome samples clustered according to host phylogenetic relationships but with weak bootstrap support (Fig. S7). Complete-linkage hierarchical clustering of individual microbiomes also mirrored host phylogenetic relationships, depending on the beta diversity metric utilized (Fig S8). Despite incomplete concordance between the host mtDNA phylogeny and gut microbiota parsimony tree, host mitochondrial genetic distance was a significant predictor of ecological distance among microbial communities (Mantel, weighted Unifrac: *r* = 0.83, *P* < 0.001; Bray-Curtis: *r* = 0.79, *P* *<* 0.001), even after controlling for dietary distance among host species (partial Mantel, weighted Unifrac: *r* = 0.76, *P* < 0.001; Bray-Curtis: *r* = 0.7, *P* < 0.001) and geographic distance among fecal sample collection sites (partial Mantel, weighted Unifrac: *r* = 0.83, *P* < 0.001; Bray-Curtis: *r* = 0.79, *P* < 0.001).

**Fig. 4:**
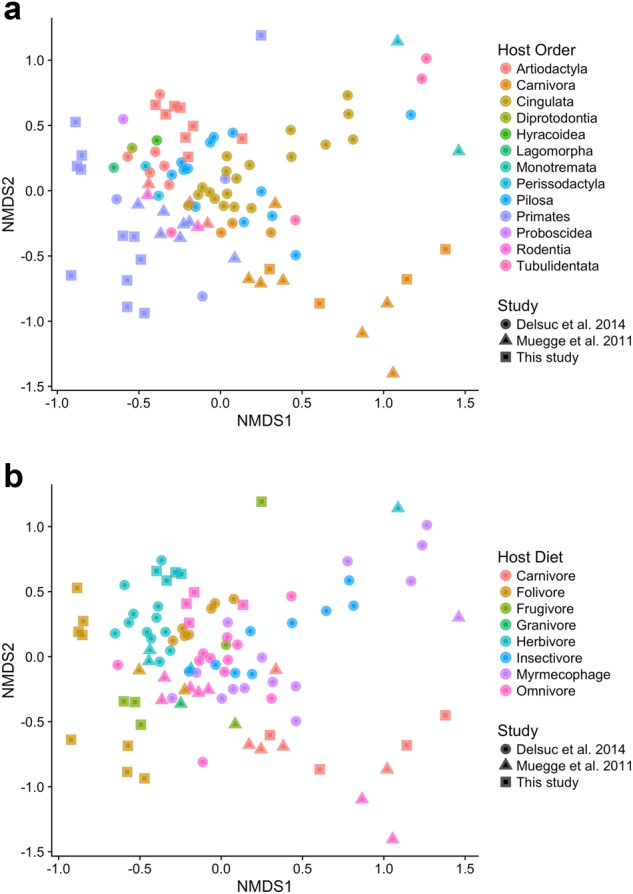
Malagasy mammal microbiomes cluster according to host order (**a**) and diet (**b**). Non-metric multidimensional scaling ordinations of family-level Bray-Curtis dissimilarities among mammalian gut microbiome samples from 52 host species across 13 orders. Samples from this study were combined with those from two other comparative studies of captive and wild animal microbiota [10, 12]

We further compared these Malagasy mammalian gut microbiomes to those of 46 other mammal species among 13 host orders [10, 12]. Bush pigs and cattle clustered with other members of Artiodactyla, lemurs with other primates, and fossa microbiomes with other carnivores (Fig. 4, S9–10). In a meta-analysis, both host order and diet contributed significantly to variation in gut microbiome composition among the 52 animal species (PERMANOVA: host order: *R*^2^ = 0.31, *P* *=* 0.001; diet: *R*^2^ = 0.24, *P* = 0.001; Fig. 4).

## Discussion

We investigated the ecological and evolutionary determinants of gut microbiome composition among six sympatric mammal species co-residing within a 1-km^2^ area in western Madagascar. As found previously [10–12, 14], variation in taxonomic composition among wild Malagasy mammals was correlated with both host evolutionary history and diet, depending on the relatedness of the host species. Moreover, shared terrestriality, but not geographic distance between sampling sites, predicted microbiome similarity among distantly related hosts with divergent diets and digestive physiologies. This suggests that ground dwelling behavior promotes the indirect horizontal transmission of commensal gut bacteria among sympatric wild mammals.

Gut bacterial diversity reflected a combination of digestive physiology, diet, and host phylogeny. The three feeding strategies represented in our study—*carnivory* and two types of herbivory, *folivory* and *frugivory*—range from foods that are energetically costly to obtain but easy to digest (i.e., animal matter), to those that are limited in quantity but fairly easy to digest (i.e., fruits and seeds), to those that are ubiquitous but difficult to digest (i.e., leaves) [67]. Carnivorous fossa and frugivorous brown lemurs require only short, simple guts because the protein and fat in animal matter and the short-chain sugars in fruit are easily assimilated using enzymes produced by the animals themselves [67, 68]. In contrast, the herbivorous and folivorous mammals (zebu cattle, sportive lemurs, and sifaka) have complex gastrointestinal tracts with lengthened digesta retention times and diverse communities of mutualistic microorganisms to facilitate fiber digestion [69, 70]. In agreement with a prior survey of 59 mammalian host species [11], we found that bush pigs and cattle, both members of the foregut-fermenting Artiodactyla order, exhibited the greatest bacterial diversity, followed by the three lemur species with intermediate diversity, and finally carnivorous fossa with lowest bacterial richness. Given that sifaka and sportive lemurs, both folivorous hindgut fermenters [71, 72], did not harbor significantly greater bacterial richness than brown lemurs, which are frugivorous with simple guts [73], we conclude that gut microbiome diversity among lemurs is shaped more by host phylogeny than by digestive physiology or diet.

The gut microbiomes of the six mammal species were distinguishable with 98.4% accuracy, and host phylogeny was the strongest predictor of compositional similarity, even when accounting for geographic proximity of sampling sites, substrate preference (i.e., ground versus tree), and dietary overlap among host species. With the exception of the lemur clade, the microbiomes of sympatric Malagasy mammal populations illustrate “phylosymbiosis,” an eco-evolutionary pattern in which the evolutionary diversification of hosts correlates with ecological changes in their microbiota [18, 74]. Congruence between host and microbial phylogenies can arise without the coevolution of entire microbial communities from a last common ancestor or co-speciation events [9, 18]. Thus, phylosymbiosis does not presuppose that all resident phylotypes in microbiomes are stable or vertically transmitted between generations [9, 18]. For example, closely related hosts with comparable behavioral traits may acquire similar bacteria from their environment or directly from other hosts [9, 75]. In mammals, phylosymbiosis has been observed in large-scale comparative studies [9], among closely related species in controlled environments [18], and in wild populations of bats [16] and apes [8, 76, 77].

In the gut microbiota parsimony tree, lemur microbiome samples clustered together separately from the other animal species but their branching order did not reflect the established evolutionary relationships among brown lemurs, sportive lemurs, and sifaka. Further, in the principal coordinate analysis, sifaka and sportive lemur microbiomes formed separate, distant clusters while brown lemur microbiomes grouped more closely with those of cattle, bush pigs, and fossa. This finding was surprising, given that sifaka and sportive lemurs are closely related species with seemingly overlapping habitat use, diet, and anatomical specializations, whereas the four semi-terrestrial and terrestrial species are distantly related with differing diets and digestive physiologies. Recent studies have found that microbiome composition and host phylogeny are strongly associated for recently diverged mammals, depending on the bacterial phylogenetic scale examined [9] and the resolution of the bacterial target gene sequenced [17]. Rather than entire gut microbial communities, specific bacterial lineages may indeed recapitulate the evolutionary relationships among lemur species [9, 22]. Further, lemur species may have diverged too recently [18] or our evolutionary model may be too simplistic to capture a strong signal of host phylogeny in the microbial community data.

The climate of western Madagascar is highly seasonal. Many Malagasy lemur species modify their diet composition and foraging strategies during the dry season as temperatures fluctuate and high quality food resources and water become scarce [73, 78]. The *Eulemur* clade are flexible frugivores [73], with high levels of folivory recorded in western populations during the dry season [79]. *Propithecus* and *Lepilemur* are both considered folivorous [71, 80–82], consuming leaves throughout the year [73, 80, 83] and fruit when available during the rainy season [43, 73, 80, 84]. We found that the predicted functional composition of lemur microbiomes reflected differences in carbohydrate and amino acid utilization, with red-fronted brown lemurs (*Eulemur rufifrons*) displaying frugivore dietary profiles, Verreaux’s sifaka (*Propithecus verreauxi*) folivore profiles, and red-tailed sportive lemurs (*Lepilemur ruficaudatus*) intermediate frugivore-folivore dietary profiles. As found for captive lemurs [85], both sportive lemur and sifaka microbiomes were enriched for folivorous pathways associated with increased plant fiber degradation, such as propanoate and butanoate metabolism [86]. Along with brown lemur microbiomes, sportive lemur microbiomes were also elevated for frugivorous pathways related to sugar metabolism and amino acid biosynthesis. Thus, frugivory may explain the gut microbial similarity between these two species, despite the more recent evolutionary divergence between sifaka and brown lemurs. Seasonal frugivory observed in captive sportive lemurs supports this claim [80]. However, there have been insufficient dietary studies of *Lepilemur* spp. in dry deciduous forests to definitively compare the fruit consumption of wild sportive lemurs and sifaka [80, 84]. Additionally, it is important to note that PICRUSt has reduced accuracy in predicting non-human metagenomes due to the bias of the Greengenes reference database towards well-studied human-associated microbes [87]. Consequently, our conclusions concerning diet-associated signals in predicted lemur gut metagenomes are speculative, especially given the high proportions of unclassified sequence reads in sportive lemur and sifaka microbiomes.

The striking discordance between sifaka and sportive lemur microbiomes may also stem from differences in host body size, activity patterns, and digestive strategies. Sifaka are diurnal with a body mass of 2.5–4 kg [44], while sportive lemurs are remarkably small-bodied (<1 kg) for a folivorous species [88]. Large body size is considered a morphological adaptation to folivory, providing increased gastrointestinal surface area and retention time for nutrient absorption [67]. Caecotrophy—re-ingestion of feces—has been observed in one sportive lemur species (*L. mustelinus)* and may be an adaptation to increase nitrogen utilization [81]. However, caecotrophy has not been observed in our study species (*L. ruficaudatus*) or other sportive lemurs [84, 88, 89]. Instead, they may manage their folivorous diet and small body size by conserving energy; they have one of the lowest basal metabolic rates among folivorous mammals [90] and long nighttime resting periods [81, 88]. Although sifaka and sportive lemurs are both hindgut fermenters, their digestive strategies lie at opposite ends of the continuum observed for primates. Sifaka employ an “efficiency” strategy, characterized by low intake, long mean retention time, and high fiber digestibility [91], while sportive lemurs use an “intake” strategy, with high intake, short mean retention time, and low fiber digestibility [68, 83]. Thus, the gut microbiota of sifaka may be better suited for degrading structural carbohydrates and detoxifying plant secondary compounds.

Finally, shared terrestriality was a significant predictor of microbial similarity, with the microbiomes of semi-terrestrial brown lemurs and fossa clustered closely to those of terrestrial bush pigs and cattle. Although a prior meta-analysis of primate parasite diversity failed to find a substrate effect [64], other studies have reported lower parasite prevalence in arboreal primates compared to sympatric terrestrial primates [92–94]. These studies did not, however, account for disparities in home range size, which may influence exposure to microorganisms [95]. Thus, terrestrial and semi-terrestrial animals may have greater exposure to fecal-orally transmitted or soil-borne gut micro-organisms and parasites compared to their arboreal counterparts [94, 95]. The arboreal lifestyles and comparatively smaller home ranges of sifaka and sportive lemurs may limit incidental contact with enteric bacteria in the environment [92, 95], and thus compound the effects of their unique diets and physiologies in driving the divergence of their gut microbial communities.

Although we focused on commensal and mutualistic gut microbiota, our findings may also apply to pathogenic bacteria that exploit similar molecular mechanisms to colonize hosts [96]. We speculate that microbiome overlap among distantly related terrestrial species is driven by exposure to heterospecific fecal material on the ground. Terrestrial animals may therefore be more vulnerable to cross-species spillover of enteric bacteria compared to arboreal animals [92, 94, 95]. A recent study [97] reported gut bacterial similarities within predator-prey host-species pairs in North America, suggesting that mammalian food chains may serve as transmission routes for gut bacteria. In contrast, sifaka and sportive lemurs constitute a high proportion of the fossa diet in western deciduous forests [98, 99], and we found that the microbiomes of these three animal species were markedly distinct.

## Conclusions and future directions

Our comparative survey of mammalian gut microbial communities is one of very few to assay cohabiting wildlife populations and is unique in its small geographic extent. By eliminating the potentially confounding effects of geography and captivity, we are able to elucidate the relative contributions of diet, behavior, and evolutionary history on gut microbial diversity in understudied Malagasy mammalian species. In contrast to prior microbiome surveys of geographically distinct or captive mammal populations [8–12, 17], we find that the gut microbiomes of distantly related animal species reflect their hosts’ evolutionary relationships rather than their dietary classifications. However, among closely related primate species, diet obscures the signal of host phylogeny in gut microbial composition. Epidemiological studies in wildlife have elucidated cross-species transmission of pathogens [64, 100], but little is known about the transfer of commensal microbes among co-occurring wild animal populations [28, 97]. More comprehensive comparative studies of wild mammal microbiomes are needed to fully resolve the dynamic dietary, physiological, social, and environmental factors that constrain gut microbiome ecology and evolution. Such insights will advance our understanding of host-microbiota interactions and the evolution of mammalian dietary flexibility and diversification and improve strategies for wildlife conservation and captive management.

### Data availability

Raw sequence data are deposited in the NCBI Sequence Read Archive under accession SRP155052.

## Electronic supplementary material

Supplementary Information
