# Supplementary material for: Terrestriality and bacterial transfer: a comparative study of gut microbiomes in sympatric Malagasy mammals
Source: ISME J. 2018 Aug 14;13(1):50–63. doi: 10.1038/s41396-018-0251-5 (PMC6299109; doi:10.1038/s41396-018-0251-5)
Supplement: Supplementary file 1 — Supplementary Information [file 41396_2018_251_MOESM1_ESM.docx]

**Supplementary Information for the article:**

Terrestriality and bacterial transfer: A comparative study of gut microbiomes in sympatric Malagasy mammals

Amanda C. Perofsky^1*^, Rebecca J. Lewis^2^, Lauren Ancel Meyers^1,3^

^1^ Department of Integrative Biology, ^2^ Department of Anthropology, The University of Texas at Austin, Austin, TX 78712, ^3^ Santa Fe Institute, Santa Fe, NM 87501

**Author for correspondence**: Amanda Perofsky [aperofsky@utexas.edu](mailto:aperofsky@utexas.edu)

**Supplemental Methods**

***Ethics statement.*** All data collection procedures complied with protocols approved by The University of Texas at Austin Institutional Animal Care and Use Committee (IACUC) and adhered to the legal requirements of Madagascar and the American Society of Primatologists (ASP) Principles for the Ethical Treatment of Non-Human Primates. Fecal samples were collected under permit approval from Madagascar National Parks (MNP), the Madagascar Ministry of Environment, Forests, and Tourism (MEFT), and the Madagascar Ministry of Higher Education.

***Fecal sample collection.*** We collected sifaka (N = 14), sportive lemur (N = 14), and brown lemur samples (N = 11) from unique individuals via focal animal follows, immediately after individuals were observed defecating. Sifaka fecal samples were collected from long-term study individuals marked with unique collars and tags. Sportive lemur samples were collected from unmarked individual animals after they emerged from their sleep tree holes at dusk. Because sportive lemurs have small home ranges (< 1 ha), we sampled unique individuals by considering only fecal samples collected from distant regions of the research site. Brown lemur samples were collected from unmarked individuals in three social groups that inhabit different sections of the research site. At the time of sample collection, we distinguished unique individuals within each brown lemur social group according to age (adult or non-adult) and sex (brown lemurs are sexually dichromatic). We opportunistically collected fossa (N = 6) and bush pig samples (N = 8) within the research site and cattle samples (N = 8) along a road that traverses Kirindy Mitea. Fossa are generally solitary and have low population densities (0.26 individuals/km^2^) (6); thus, fossa fecal samples potentially originated from one or a few individuals. Although bush pigs in Madagascar are understudied, bush pigs in South Africa are known to live in territorial sounders (groups) of up to twelve individuals, and their population densities are 0.3—0.5 individuals/ km^2^ (5). Thus, the bushpig samples in our study potentially originated from several different individuals from one sounder. Zebu cattle samples were collected along a road with frequent foot traffic, with 5—10 zebu carts/day (Lewis, personal observation). Thus, our cattle fecal samples likely originated from different animals.

***Host phylogenetic relationships.*** We estimated phylogenetic distances among mammal species using mitochondrial cytochrome b (cyt-b) gene sequences downloaded from NCBI and aligned using Muscle 3.8.31 (21). We evaluated models of molecular evolution (*phangorn* package) (22) and then reconstructed maximum likelihood phylogenetic trees based on the ‘TIM2+G’ model and 1000 bootstrap replications.

***Dietary distance among host species.*** We computed a dietary distance matrix for the six mammal species based on the proportions of dietary items consumed by each species (methods in supplementary information). Following the EltonTraits database classification system (23), we defined ten dietary items (Invertebrates, Vertebrates excluding fishes, Reptiles/Amphibians, Carrion, Fish, Unknown Vertebrates, Fruits, Nectar/Pollen/Gums, Seeds/Nuts, Leaves/Grasses/Ground Vegetation), and each species was assigned a percentage for each dietary item. We then used Euclidean distances to compute a dietary distance matrix for the six species based on the proportions of dietary items consumed by each species.

***Microbiome OTU-based analyses*.** We generated a closed-reference 97% Operational Taxonomic Unit (OTU) dataset for use in two downstream analyses. First, we demultiplexed and quality filtered raw Illumina sequence reads using QIIME (24) with default settings. We assigned reads to 97% OTUs using the *uclust* algorithm, excluded chimeras with ChimeraSlayer, and assigned taxonomic classifications with the RDP classifier based on their best match in the Greengenes database (13_5 release). We then removed singletons, OTUs designated as ‘unclassified’ or ‘Eukaryota’ at the kingdom level, and OTUs annotated as chloroplast or mitochondria to generate a usable OTU table.

**Mammalian microbiome meta-analysis.** We combined our 97% OTU dataset with two previously published 97% OTU microbiome datasets encompassing 47 mammal species across 13 orders (25,26). We assembled a closed-reference 97% OTU comparative dataset with a total of 101 samples (up to four samples per mammal species chosen at random) and removed OTUs with less than 0.000001 relative abundance. This combined dataset included 21 samples from Muegge *et al*.’s study targeting a different 16S region (V2) and sequenced on the 454 platform (26). Following the protocol of Delsuc *et al*.’s study (25), we rarefied the comparative dataset to 1100 sequences per sample and constructed an OTU table at the bacterial family level to diminish biases in sequencing technology and 16S target region. We used non-metric multidimensional scaling to visualize Bray-Curtis dissimilarities among microbiome samples.

**Metagenomic predictions based on 16S rRNA sequencing data.** We used PICRUSt (phylogenetic investigation of communities by reconstruction of unobserved states) v1.1.1 (27) to generate metagenomic functional profiles of lemur gut microbiomes based on patterns observed in 16S-based 97% OTU microbial community profiles. PICRUSt uses an extended ancestral-state algorithm to predict gene family abundances (*i.e.*, KEGG orthologs or pathways) from 16S data and the Greengenes reference genome database. To visualize similarities in functional profiles among lemur species, we used redundancy analyses to construct principal component ordinations of KEGG orthologs and pathways. We created ordinations for the dataset as a whole (*i.e.*, all KEGG orthologs), as well as for individual KEGG sub-pathways related to carbohydrate and essential amino acid metabolism. We implemented an RFC model to determine the degree to which individual predicted metagenomes can be classified into their respective host species.

***Microbiome composition and host phylogeny.*** We used a parsimony approach (28) to determine whether similarities in gut microbiome composition are congruent with the evolutionary relationships among mammalian host species First, we randomly selected six microbiome samples from each host species and pruned the table of reproducible ASVs to include only these samples (N_samples_ = 36). Because unique bacterial phylotypes cannot provide information about the relatedness among samples, we limited our phylogenetic analysis to ASVs detected in two or more samples, which included 2323 ASVs. We then collapsed the ASV table by host species to obtain representative microbiome profiles. To construct a host species-level microbiome phylogenetic tree, we transformed the collapsed ASV table into a six-state 2323-character matrix, with each character corresponding to an ASV whose absolute abundance was normalized by coding into one of six states reflecting log-unit differences in occurrence, with ‘1’ corresponding to an ASV’s absence in a given sample. Characters were ordered, such that transitions between distant states (*i.e.*, samples having very divergent frequencies of a particular ASV) were costlier than between similar states. We then subjected the six-state character matrix to a heuristic maximum parsimony tree search (*phangorn* package) (22), with 1000 pseudo-replicates used to assess bootstrap support. We repeated this analysis using the original ASV table, in which samples were not collapsed according to host species (Fig. S7). We assessed congruence between the host phylogeny from host mitochondrial cytochrome b data and the microbiome-based tree by calculating a normalized Robinson-Foulds score (*phangorn* package) (22), which divides the distance between two tree topologies by the maximum possible distance.

***Congruence between host and gut microbiome topologies.*** We assessed congruence between the host phylogeny from host mitochondrial cytochrome b data and the microbiome-based tree by calculating a normalized Robinson-Foulds score (*phangorn* package) (22), which divides the distance between two tree topologies by the maximum possible distance. To assess whether the host and microbiome trees were more topologically congruent than expected by chance, we simulated 100,000 sets of stochastic, uncorrelated trees with labels matching the original host and microbiome trees (29). We determined the Robinson-Foulds *p-*value by the probability of 100,000 randomized sets of trees yielding equivalent or more congruent topologies than our host and microbiome trees.

***Predictors of pairwise similarity in microbiome taxonomic composition.*** We used a linear mixed-effect models to fit weighted Unifrac distances among 1830 conspecific and interspecific pairs to potential *pairwise* predictors of similarity in microbiome composition. Because weighted Unifrac distances are continuous proportions, we fit mixed-effect Beta regression models to the microbial data using the *glmmADMB* package (30). The variables included in model selection were dietary distance, geographic distance, mtDNA phylogenetic distance, and shared terrestriality (‘yes’ versus ‘no’). Each microbiome sample could appear interchangeably as individual A or individual B in a pairwise comparison; thus, we controlled for autocorrelation by modeling the identities of individual samples in each pair as random effects.

**Supplemental References**

1.	Chivers DJ, Langer P, editors. The Digestive System in Mammals. Cambridge: Cambridge University Press; 1994.

2.	Ratovonamana RY, Rajeriarison C, Roger E, Kiefer I, Ganzhorn JU. Impact of livestock grazing on forest structure, plant species composition and biomass in Southwestern Madagascar. Scr Bot Belgica. 2013;50:82–92.

3.	Melton DA, Cooper SM, Whittington AE. The diet of bushpigs in a sugar-cane agroecosystem. South African J Wildl Res. 1989;19(1):48–51.

4.	Ghiglieri MP, Butynski TM, Struhsaker TT, Leland L, Wallis SJ, Waser P. Bush pig (*Potamochoerus porcus*) polychromatism and ecology in Kibale Forest, Uganda. Afr J Ecol. 1982;20(4):231–6.

5.	Venter J, Ehlers-Smith Y, Seydeck A. A Conservation Assessment of Potamochoerus larvatus. In: Child M, Roxburgh L, Do Linh San E, Raimondo D, Davies-Mostert H, editors. The Red List of Mammals of South Africa, Swaziland and Lesotho. South Africa: South African National Biodiversity Institute and Endangered Wildlife Trust; 2016.

6.	Hawkins CE, Racey PA. Food Habits of an Endangered Carnivore, Cryptoprocta ferox, in the Dry Deciduous Forests of Western Madagascar. J Mammal. 2008;89(1):64–74.

7.	Dollar L, Ganzhorn JU, Goodman SM. Primates and Other Prey in the Seasonally Variable Diet of *Cryptoprocta ferox* in the Dry Deciduous Forest of Western Madagascar. In: Gursky SL, Nekaris KAI, editors. Primate Anti-Predator Strategies. Boston, MA: Springer; 2007. p. 63–76.

8.	Hawkins CE, Racey PA. Low population density of a tropical forest carnivore, *Cryptoprocta ferox*: Implications for protected area management. Oryx. 2005;39(1):35–43.

9.	Campbell JL, Eisemann JH, Williams C V, Glenn KM. Description of the gastrointestinal tract of five Lemur species: *Propithecus tattersalli, Propithecus verreauxi coquereli, Varecia variegata, Hapalemur griseus,* and *Lemur catta*. Am J Primatol. 2000;52(3):133–42.

10.	Campbell JL, Williams C V., Eisemann JH. Use of total dietary fiber across four lemur species (*Propithecus verreauxi coquereli, Hapalemur griseus griseus, Varecia variegata,* and *Eulemur fulvus*): Does fiber type affect digestive efficiency? Am J Primatol. 2004;64(3):323–35.

11.	Lewis RJ, Kappeler PM. Seasonality, body condition, and timing of reproduction in Propithecus verreauxi verreauxi in the Kirindy Forest. Am J Primatol. 2005;67(3):347–64.

12.	Loudon JE, Sauther ML. Verreaux’s sifaka (*Propithecus verreauxi*) and ring-tailed lemur (*Lemur catta*) endoparasitism at the Bezà Mahafaly Special Reserve. Madagascar Conserv Dev. 2013;8(1):21–8.

13.	Perofsky AC, Lewis RJ, Abondano LA, Di Fiore A, Meyers LA. Hierarchical social networks shape gut microbial composition in wild Verreaux’s sifaka. Proc R Soc B. 2017;284(20172274).

14.	Leimberger KG, Lewis RJ. Patterns of male dispersal in Verreaux’s sifaka (*Propithecus verreauxi*) at Kirindy Mitea National Park. Am J Primatol. 2015;1098–2345.

15.	Hladik CM, Charles-Dominique P, Petter J-J. Feeding Strategies of Five Nocturnal Prosimians in the Dry Forest of the West Coast of Madagascar. In: Nocturnal Malagasy Primates Ecology, Physiology, and Behavior. 1980. p. 41–73.

16.	Zinner D, Hilgartner RD, Kappeler PM, Pietsch T. Social Organization of Lepilemur ruficaudatus. Int J Primatol. 2003;24(4):869–88.

17.	Lambert JE. Primate digestion: Interactions among anatomy, physiology, and feeding ecology. Evol Anthropol. 1998;7(1):8–20.

18.	Ganzhorn JU. Distribution of a folivorous lemur in relation to seasonally varying food resources: Integrating quantitative and qualitative aspects of food characteristics. Oecologia. 2002;131(3):427–35.

19.	Donati G, Lunardini A, Kappeler PM. Cathemeral Activity of Red-Fronted Brown Lemurs (*Eulemur Fulvus Rufus*) in the Kirindy Forest/CFPF. In: Rakotosamimanana B, Rasamimanana H, Ganzhorn JU, Goodman SM, editors. New Directions in Lemur Studies. Boston, MA: Springer US; 1999. p. 119–37.

20.	Sato H, Santini L, Patel ER, Campera M, Yamashita N, Colquhoun IC, et al. Dietary Flexibility and Feeding Strategies of Eulemur: A Comparison with Propithecus. Int J Primatol. 2016;37(1):109–29.

21.	Edgar RC. MUSCLE: Multiple sequence alignment with high accuracy and high throughput. Nucleic Acids Res. 2004;32(5):1792–7.

22.	Schliep KP. phangorn: Phylogenetic analysis in R. Bioinformatics. 2011;27(4):592–3.

23.	Wilman H, Belmaker J, Simpson J, de la Rosa C, Rivadeneira MM, Jetz W. EltonTraits 1.0: Species-level foraging attributes of the world’s birds and mammals. Ecology. 2014;95(7):2027.

24.	Caporaso JG, Kuczynski J, Stombaugh J, Bittinger K, Bushman FD, Costello EK, et al. QIIME allows analysis of high-throughput community sequencing data. Nat Methods. 2010;7(5):335–6.

25.	Delsuc F, Metcalf JL, Wegener Parfrey L, Song SJ, González A, Knight R. Convergence of gut microbiomes in myrmecophagous mammals. Mol Ecol. 2014;23(6):1301–17.

26.	Muegge B, Kuczynski J, Knights D. Diet drives convergence in gut microbiome functions across mammalian phylogeny and within humans. Science (80- ). 2011;332:970–4.

27.	Langille MGI, Zaneveld J, Caporaso JG, McDonald D, Knights D, Reyes JA, et al. Predictive functional profiling of microbial communities using 16S rRNA marker gene sequences. Nat Biotechnol. 2013;31(9):814–21.

28.	Ochman H, Worobey M, Kuo C-H, Ndjango J-BN, Peeters M, Hahn BH, et al. Evolutionary relationships of wild hominids recapitulated by gut microbial communities. PLoS Biol. 2010;8(11):e1000546.

29.	Revell LJ. phytools: An R package for phylogenetic comparative biology (and other things). Methods Ecol Evol. 2012;3(2):217–23.

30.	Skaug H, Fournier D, Nielsen A, Magnusson A, Bolker B. glmmADMB: Generalized Linear Mixed Models using “AD Model Builder.” 2016.

**Supplemental Figures and Tables**

**Figure S1. Map indicating the location of Ankoatsifaka Research Station.** Ankoatsifaka Research Station (20°47.69′S, 44°9.88′E) is located in a terrestrial section of Kirindy Mitea UNESCO Biosphere, near the western coast of Madagascar.

**Figure S2. Heatmap of the 50 most discriminative bacteria genera in distinguishing gut microbiomes among six sympatric mammal species.** Differential abundance of bacterial genera among animal species was assessed using the non-parametric SAMseq method.

Bush pig

Brown lemur

Fossa

Sportive lemur

Verreaux’s sifaka

Zebu cattle

**Figure S3. Gut microbial diversity among six sympatric mammal species.** Mean observed richness (number of ASVs) and Shannon’s diversity were calculated from 100 ASV tables rarefied to 31,191 reads for each sample. Differences among mammal species were evaluated using Kruskal-Wallis tests adjusted for multiple comparisons.

**Figure S4. Average silhouette width values for partitioning around medoids (PAM) clustering with different numbers of clusters (k).** Five (clusters) was chosen as the optimal number. This analysis is based weighted Unifrac distances (left) and Bray-Curtis dissimilarities (right) among 61 microbiome samples from six mammal species.

**Figure S5. Principal components analysis ordinations of predicted metagenomic functional potential in brown lemur, sportive lemur, and Verreaux’s sifaka microbiomes.** Lemur microbiomes cluster according to host species identity when considering all KEGG pathways (A) or sub-pathways involved in carbohydrate metabolism (B) and amino acid metabolism (C). Metagenomic functional profiles were inferred using PICRUSt (phylogenetic investigation of communities by reconstruction of unobserved states).

**Figure S6. Heatmap of predicted amino acid metabolism in brown lemur, sportive lemur, and Verreaux’s sifaka gut microbiomes.** The transformed relative abundances of the 19 most abundant amino acid KEGG sub-pathways are shown, with the white color representing the relative abundance of pathways having the column average, blue tones indicating relative abundances less than the column average, and red tones indicating relative abundances greater than the column average. Metagenomic functional profiles were inferred using PICRUSt (phylogenetic investigation of communities by reconstruction of unobserved states).

**Figure S7. Comparison between gut microbiota tree and host phylogeny relationships.** The maximum parsimony gut microbiota tree (left) is based on the abundances of amplicon sequence variants in 61 microbiome samples. The maximum likelihood host species phylogeny (right) is based mitochondrial cytochrome b (cyt-b) gene sequences. The host phylogeny scale bar indicates nucleotide substitutions per site.

**Figure S8.** Dendrograms obtained from complete linkage hierarchical clustering of Bray-Curtis dissimilarities (A) and weighted Unifrac distances (B) among 61 microbiome samples.

**Figure S9. Malagasy mammal microbiomes cluster according to host order.** Non-metric multidimensional scaling ordinations of family-level Bray-Curtis dissimilarities among mammalian gut microbiome samples from 52 host species across 13 orders. Individual samples are labeled according to host species common name. Samples from this study were combined with those from two other comparative studies of captive and wild animal microbiota (25,26).

**Figure S10. Malagasy mammal microbiomes cluster according to host order.** Non-metric multidimensional scaling (NMDS) ordination of family-level Bray-Curtis dissimilarities among 97% OTU microbiome samples collected from 30 host species across the three mammal orders included in this study (Artiodactyla, Carnivora, Primates). Malagasy samples from this study were combined with two other comparative studies of captive and wild animal gut microbiota (25,26).

**Table S1. Sample metadata (GPS locations available by request)**

| **Sample ID** | **Animal Species** | **Collection date** |
| --- | --- | --- |
| B3 | Brown lemur | 6/25/12 |
| B6 | Brown lemur | 7/3/12 |
| B1 | Brown lemur | 7/16/12 |
| B8 | Brown lemur | 7/24/12 |
| B9 | Brown lemur | 7/24/12 |
| B11 | Brown lemur | 7/25/12 |
| B5 | Brown lemur | 7/25/12 |
| B7 | Brown lemur | 7/25/12 |
| B12 | Brown lemur | 7/30/12 |
| B4 | Brown lemur | 7/31/12 |
| B13 | Brown lemur | 8/1/12 |
| BP16 | Bush pig | 6/10/12 |
| BP15 | Bush pig | 6/14/12 |
| BP9 | Bush pig | 6/18/12 |
| BP11 | Bush pig | 6/21/12 |
| BP13 | Bush pig | 6/25/12 |
| BP14 | Bush pig | 7/12/12 |
| BP17 | Bush pig | 7/14/12 |
| BP10 | Bush pig | 7/23/12 |
| F18 | Fossa | 6/30/12 |
| F22 | Fossa | 6/30/12 |
| F19 | Fossa | 7/1/12 |
| F23 | Fossa | 7/2/12 |
| F24 | Fossa | 7/14/12 |
| F20 | Fossa | 7/24/12 |
| LS1 | Sifaka | 6/20/12 |
| ZF1 | Sifaka | 6/23/12 |
| HP4 | Sifaka | 6/24/12 |
| VC5 | Sifaka | 7/3/12 |
| AN5 | Sifaka | 7/4/12 |
| AP5 | Sifaka | 7/4/12 |
| PM6 | Sifaka | 7/9/12 |
| JU4 | Sifaka | 7/10/12 |
| JK6 | Sifaka | 7/15/12 |
| AB8 | Sifaka | 7/19/12 |
| NC3 | Sifaka | 7/20/12 |
| HS10 | Sifaka | 7/24/12 |
| VR13 | Sifaka | 7/24/12 |
| IC9 | Sifaka | 7/25/12 |
| L32 | Sportive lemur | 7/11/12 |
| L35 | Sportive lemur | 7/11/12 |
| L40 | Sportive lemur | 7/11/12 |
| L15 | Sportive lemur | 7/15/12 |
| L20 | Sportive lemur | 7/15/12 |
| L1 | Sportive lemur | 7/16/12 |
| L13 | Sportive lemur | 7/17/12 |
| L39 | Sportive lemur | 7/19/12 |
| L9 | Sportive lemur | 7/19/12 |
| L18 | Sportive lemur | 7/21/12 |
| L23 | Sportive lemur | 7/23/12 |
| L6 | Sportive lemur | 7/26/12 |
| L31 | Sportive lemur | 7/30/12 |
| L5 | Sportive lemur | 7/31/12 |
| Z8 | Zebu cattle | 6/13/12 |
| Z5 | Zebu cattle | 6/17/12 |
| Z2 | Zebu cattle | 6/22/12 |
| Z3 | Zebu cattle | 6/22/12 |
| Z1 | Zebu cattle | 6/30/12 |
| Z6 | Zebu cattle | 6/30/12 |
| Z4 | Zebu cattle | 7/14/12 |
| Z7 | Zebu cattle | 7/14/12 |

**Table S2. Differentially abundant bacterial phyla, families, and genera among six sympatric mammal species inhabiting Kirindy Mitea Biosphere, Madagascar.** For each significant bacterial taxon, the ranking score, the contrast in each animal species (the standardized mean difference between the taxon’s abundance in that species versus its overall mean abundance), and the FDR-adjusted *P* value are shown.

| **Phylum** | **Score** | **Fossa** | **Sportive lemur** | **Verreaux’s sifaka** | **Brown lemur** | **Bush pig** | **Zebu cattle** | ***P*** |
| --- | --- | --- | --- | --- | --- | --- | --- | --- |
| Unclassified | 53.148 | -3.829 | 5.643 | 1.912 | -3.473 | -0.357 | -1.722 | < 0.0001 |
| Synergistetes | 53.004 | -2.678 | 5.597 | 2.029 | -0.503 | -3.274 | -3.292 | < 0.0001 |
| Spirochaetes | 47.701 | -2.559 | -4.14 | -1.911 | 3.2 | 3.698 | 2.453 | < 0.0001 |
| Verrucomicrobia | 47.465 | -2.649 | 3.816 | 3.616 | -3.653 | -3.117 | 0.356 | < 0.0001 |
| Kiritimatiellaeota | 46.545 | -2.045 | -3.182 | -3.242 | 4.016 | 3.383 | 1.849 | < 0.0001 |
| Actinobacteria | 46.395 | 3.375 | 4.051 | 0.283 | -1.524 | -2.156 | -4.484 | < 0.0001 |
| Euryarchaeota | 42.058 | -2.771 | 2.157 | -4.71 | 2.713 | 3.042 | -0.507 | < 0.0001 |
| Firmicutes | 41.178 | 3.576 | 1.26 | -3.446 | -3.743 | 2.817 | 1.014 | < 0.0001 |
| Cyanobacteria | 40.43 | -3.859 | -3.125 | 4.084 | 2.695 | -0.86 | 0.001 | < 0.0001 |
| Epsilonbacteraeota | 40.383 | 1.896 | -3.382 | 3.825 | 2.509 | -2.509 | -2.573 | < 0.0001 |
| Tenericutes | 39.069 | -3.932 | 0.103 | -3.026 | 0.703 | 3.607 | 2.702 | < 0.0001 |
| Fibrobacteres | 37.665 | -1.75 | -2.65 | 5.63 | -2.017 | 0.62 | -0.491 | < 0.0001 |
| Bacteroidetes | 36.399 | -2.742 | -3.639 | 1.875 | 0.755 | -0.533 | 4.289 | < 0.0001 |
| Lentisphaerae | 31.996 | -1.371 | -1.256 | -2.099 | -1.551 | 3.455 | 3.7 | < 0.0001 |
| Proteobacteria | 29.864 | 1.589 | -5.112 | 2.569 | 0.386 | 1.584 | -0.258 | < 0.0001 |
| Planctomycetes | 28.305 | -1.09 | -1.618 | -1.687 | -1.382 | 3.789 | 2.863 | < 0.0001 |
| Fusobacteria | 14.762 | 3.292 | -0.428 | -0.685 | -0.572 | -0.46 | -0.407 | < 0.0001 |
| **Family** | **Score** | **Fossa** | **Sportive lemur** | **Verreaux’s sifaka** | **Brown lemur** | **Bush pig** | **Zebu cattle** | ***P*** |
| **Actinobacteria** |  |  |  |  |  |  |  |  |
| Unclassified Coriobacteriales | 48.547 | -2.789 | -2.47 | 5.259 | -3.633 | 1.93 | 1.195 | < 0.0001 |
| Coriobacteriales-Atopobiaceae | 39.456 | -1.447 | 5.584 | -2.413 | -2.085 | -1.676 | 1.376 | < 0.0001 |
| Coriobacteriales-Coriobacteriaceae | 36.564 | 1.593 | -5.347 | 2.364 | 2.74 | 0.516 | -1.326 | < 0.0001 |
| Coriobacteriales-Eggerthellaceae | 35.244 | 0.556 | 4.639 | -3.176 | 1.646 | -1.433 | -2.755 | < 0.0001 |
| Bifidobacteriales-Bifidobacteriaceae | 33.921 | -1.252 | -2.255 | 0.989 | 5.159 | -1.616 | -1.578 | < 0.0001 |
| Coriobacteriales-Coriobacteriales Incertae Sedis | 29.961 | -1.479 | 1.681 | -2.594 | -2.089 | 0.621 | 4.201 | < 0.0001 |
| Pseudonocardiales-Pseudonocardiaceae | 23.606 | -0.7 | -0.825 | -0.971 | -0.897 | -0.653 | 4.529 | < 0.0001 |
| Actinomycetales-Actinomycetaceae | 16.217 | -0.979 | 1.883 | -1.686 | 2.644 | -1.075 | -1.319 | < 0.0001 |
| Streptomycetales-Streptomycetaceae | 12.137 | -0.363 | -0.526 | -0.735 | -0.429 | 2.857 | -0.478 | < 0.0001 |
| Solirubrobacterales-Solirubrobacteraceae | 11.342 | -0.398 | -0.807 | 2.792 | -0.716 | -0.74 | -0.566 | < 0.0001 |
| Corynebacteriales-Corynebacteriaceae | 7.285 | -0.427 | -0.306 | -0.425 | -0.177 | 1.757 | -0.267 | < 0.0001 |
| **Bacteroidetes** |  |  |  |  |  |  |  |  |
| Bacteroidales-Bacteroidaceae | 46.959 | 3.65 | 2.454 | -1.977 | -5.09 | 2.573 | -0.589 | < 0.0001 |
| Bacteroidales-Barnesiellaceae | 45.022 | -2.442 | 5.172 | -4.238 | 1.861 | 0.362 | -1.49 | < 0.0001 |
| Bacteroidales-Tannerellaceae | 44.974 | -3.13 | -3.665 | 3.564 | 0.076 | -1.279 | 4.081 | < 0.0001 |
| Bacteroidales-Bacteroidales RF16 group | 44.61 | -3.004 | 0.443 | 5.174 | -3.56 | -1.852 | 1.559 | < 0.0001 |
| Bacteroidales-Muribaculaceae | 43.052 | -3.638 | 0.331 | 4.326 | -4.174 | 1.471 | 0.691 | < 0.0001 |
| Bacteroidales-Unclassified | 41.051 | 0.557 | 5.039 | -0.627 | -4.776 | 0.817 | -1.365 | < 0.0001 |
| Bacteroidales-Marinifilaceae | 36.034 | -1.83 | 2.109 | -3.145 | 0.788 | -2.295 | 4.302 | < 0.0001 |
| Bacteroidales-Paludibacteraceae | 35.894 | -2.507 | 5.005 | -0.621 | -3.488 | 0.462 | 0.261 | < 0.0001 |
| Bacteroidales-Prevotellaceae | 29.802 | 0.208 | 4.767 | -1.674 | -0.223 | -3.373 | -0.409 | < 0.0001 |
| Unclassified Flavobacteriales | 26.615 | -1.171 | -1.821 | 4.777 | -1.464 | -1.246 | 0.264 | < 0.0001 |
| Bacteroidales-F082 | 26.013 | -0.969 | 4.81 | -1.309 | -1.418 | -0.974 | -0.916 | < 0.0001 |
| Bacteroidales-Bacteroidales BS11 gut group | 23.767 | -0.424 | -1.045 | -0.962 | -0.77 | -0.778 | 4.529 | < 0.0001 |
| Bacteroidales-Rikenellaceae | 17.583 | 2.707 | 0.492 | -1.225 | 0.459 | 1.109 | -3.107 | < 0.0001 |
| Bacteroidales-p-2534-18B5 gut group | 15.968 | -0.586 | -1.039 | -1.101 | -0.902 | 3.318 | 0.891 | < 0.0001 |
| Flavobacteriales-Flavobacteriaceae | 14.034 | -0.55 | 3.232 | -0.994 | -0.911 | -0.654 | -0.611 | < 0.0001 |
| Bacteroidales-p-251-o5 | 7.155 | -0.243 | -0.404 | -0.213 | -0.455 | -0.187 | 1.688 | < 0.0001 |
| Bacteroidales-  Bacteroidales UCG-001 | 5.627 | -0.162 | -0.327 | 1.231 | -0.301 | -0.317 | -0.325 | < 0.0001 |
| **Cyanobacteria** |  | | | | | | | |
| Unclassified Gastranaerophilales | 45.088 | 0.882 | 4.78 | -5.365 | -1.837 | 1.14 | 0.902 | < 0.0001 |
| **Elusimicrobia** |  |  |  |  |  |  |  |  |
| Elusimicrobiales-Elusimicrobiaceae | 41.359 | -1.815 | 5.544 | -3.041 | -2.56 | 0.245 | 1.152 | < 0.0001 |
| **Epsilonbacteraeota** |  | | | | | | | |
| Campylobacterales-Helicobacteraceae | 24.75 | -0.496 | -2.082 | 3.551 | -1.892 | -1.555 | 2.318 | < 0.0001 |
| Campylobacterales-Campylobacteraceae | 6.511 | -0.434 | 0.815 | -0.613 | -0.744 | -0.369 | 1.347 | < 0.0001 |
| **Euryarchaeota** |  |  |  |  |  |  |  |  |
| Methanomassiliicoccales-Methanomethylophilaceae | 42.138 | -2.557 | 0.91 | -4.166 | 5.154 | 0.074 | 0.368 | < 0.0001 |
| Methanobacteriales-Methanobacteriaceae | 40.545 | -2.839 | -1.278 | -0.297 | 4.264 | -3.298 | 2.908 | < 0.0001 |
| Methanomicrobiales-Methanocorpusculaceae | 9.438 | 1.932 | -0.752 | -0.604 | -0.678 | 1.162 | -0.406 | < 0.0001 |
| **Fibrobacteres** |  | | | | | | | |
| Fibrobacterales-Fibrobacteraceae | 22.405 | -1.377 | -1.994 | -2.213 | 2.63 | 0.754 | 2.707 | < 0.0001 |
| **Firmicutes** |  |  |  |  |  |  |  |  |
| Clostridiales-Clostridiales_vadinBB60_group | 47.625 | -3.766 | -0.404 | -4.122 | 3.901 | 1.877 | 2.641 | < 0.0001 |
| Clostridiales-Family_XIII | 45.356 | -3.996 | -0.606 | 0.392 | -3.049 | 3.7 | 3.563 | < 0.0001 |
| Clostridiales-Clostridiaceae_1 | 41.417 | -3.561 | 2.506 | 1.376 | -4.039 | 3.402 | -0.496 | < 0.0001 |
| Selenomonadales-Acidaminococcaceae | 38.352 | -1.213 | -2.708 | 5.285 | 1.511 | -1.215 | -2.647 | < 0.0001 |
| Bacillales-Planococcaceae | 36.76 | 2.22 | 4.571 | -2.569 | -1.138 | -3.382 | 0.226 | < 0.0001 |
| Unclassified Clostridiales | 35.315 | -3.112 | -0.965 | -1.804 | 5.058 | -0.931 | 1.365 | < 0.0001 |
| Clostridiales-Peptostreptococcaceae | 32.061 | 2.409 | -1.992 | 2.774 | -4.3 | 1.753 | 0.045 | < 0.0001 |
| Clostridiales-Lachnospiraceae | 30.859 | 3.996 | 0.557 | -4.385 | 1.087 | -0.496 | 0.502 | < 0.0001 |
| Erysipelotrichales-Erysipelotrichaceae | 30.454 | -3.913 | -1.863 | 4.231 | -0.089 | 0.262 | 0.341 | < 0.0001 |
| Clostridiales-Defluviitaleaceae | 30.266 | -1.757 | -0.001 | -1.544 | -2.347 | 1.624 | 4.525 | < 0.0001 |
| Lactobacillales-Enterococcaceae | 29.623 | -1.074 | 5.225 | -1.609 | -1.123 | -1.132 | -1.146 | < 0.0001 |
| Lactobacillales-Streptococcaceae | 28.92 | -1.868 | -3.113 | 2.684 | 2.233 | 1.926 | -2.287 | < 0.0001 |
| Selenomonadales-Veillonellaceae | 25.064 | -1.498 | -2.501 | 0.49 | -0.669 | 0.25 | 4.339 | < 0.0001 |
| Lactobacillales-Lactobacillaceae | 23.242 | -0.791 | -1.501 | -1.617 | 4.171 | -1.261 | 1.094 | < 0.0001 |
| Bacillales-Staphylococcaceae | 20.981 | -0.535 | -1.057 | -1.041 | 4.26 | -0.892 | -0.875 | < 0.0001 |
| Bacillales-Bacillaceae | 18.066 | -1.044 | 1.35 | -1.708 | 3.269 | -1.22 | -1.136 | < 0.0001 |
| Clostridiales-Ruminococcaceae | 15.847 | 3.51 | -0.383 | -0.498 | -1.144 | 0.997 | -1.694 | < 0.0001 |
| Clostridiales-Peptococcaceae | 15.285 | -0.79 | 0.248 | -1.194 | -1.077 | 3.366 | -0.263 | < 0.0001 |
| Clostridiales-Eubacteriaceae | 14.864 | -1.194 | 1.203 | -1.809 | -1.51 | 1.126 | 2.402 | < 0.0001 |
| Clostridiales-Christensenellaceae | 12.244 | -1.466 | -0.566 | 2.922 | -1.903 | 0.734 | -0.208 | < 0.0001 |
| Clostridiales-Family_XI | 8.167 | -0.36 | -0.551 | 2.052 | -0.229 | -0.601 | -0.691 | < 0.0001 |
| **Fusobacteria** |  |  |  |  |  |  |  |  |
| Fusobacteriales-Fusobacteriaceae | 35.263 | -1.366 | 5.171 | -2.328 | -1.97 | -1.587 | 1.494 | < 0.0001 |
| **Kiritimatiellaeota** |  | | | | | | | |
| Unclassified WCHB1-41 | 35.939 | 3.996 | 2.871 | -4.173 | -0.568 | -1.313 | 0.057 | < 0.0001 |
| **Lentisphaerae** |  |  |  |  |  |  |  |  |
| Victivallales-Victivallaceae | 40.306 | -1.83 | -2.971 | 4.564 | -2.625 | -0.353 | 2.973 | < 0.0001 |
| Oligosphaerales-Oligosphaeraceae | 30.021 | -0.56 | 2.349 | -2.352 | -2.076 | 4.126 | -1.263 | < 0.0001 |
| Victivallales-vadinBE97 | 21.689 | -0.944 | -1.667 | -1.494 | 2.188 | -1.068 | 3.348 | < 0.0001 |
| **Planctomycetes** |  |  |  |  |  |  |  |  |
| Pirellulales-Pirellulaceae | 7.517 | -0.316 | 0.821 | -0.9 | -0.718 | 1.571 | -0.376 | < 0.0001 |
| **Patescibacteria** |  | | | | | | | |
| Saccharimonadales-Saccharimonadaceae | 11.525 | -0.35 | -0.656 | -0.606 | -0.459 | 2.807 | -0.403 | < 0.0001 |
| **Proteobacteria** |  |  |  |  |  |  |  |  |
| Pseudomonadales-Pseudomonadaceae | 44.628 | -1.655 | 5.643 | -2.644 | -2.186 | -1.922 | 2.137 | < 0.0001 |
| Desulfovibrionales-Desulfovibrionaceae | 43.578 | 3.996 | 0.782 | -5.085 | -1.222 | 2.93 | 0.297 | < 0.0001 |
| Aeromonadales-Succinivibrionaceae | 42.004 | -3.353 | 4.376 | -0.524 | -2.967 | 2.989 | -1.45 | < 0.0001 |
| Betaproteobacteriales-Burkholderiaceae | 40.016 | -3.769 | 2.955 | 3.838 | -2.918 | -1.537 | -0.278 | < 0.0001 |
| Unclassified Rhodospirillales | 39.926 | -3.099 | 0.919 | 4.842 | -3.126 | -1.973 | 1.09 | < 0.0001 |
| Enterobacteriales-Enterobacteriaceae | 29.023 | -0.51 | -2.314 | 2.637 | -1.964 | -1.521 | 3.804 | < 0.0001 |
| Acetobacterales-Acetobacteraceae | 28.718 | -1.358 | 4.908 | -2.209 | -1.93 | 0.273 | -0.24 | < 0.0001 |
| Pseudomonadales-Moraxellaceae | 27.482 | -0.932 | 4.847 | -1.846 | -1.596 | -1.383 | 0.285 | < 0.0001 |
| Unclassified Betaproteobacteriales | 25.776 | -0.849 | 4.837 | -1.552 | -1.227 | -0.952 | -0.994 | < 0.0001 |
| Rhizobiales-Beijerinckiaceae | 14.599 | -0.453 | -0.853 | -0.542 | -0.739 | 3.385 | -0.406 | < 0.0001 |
| Rhodobacterales-Rhodobacteraceae | 12.332 | -0.351 | -0.643 | -0.672 | -0.537 | -0.341 | 2.9 | < 0.0001 |
| Pasteurellales-Pasteurellaceae | 11.842 | -0.75 | -1.009 | -1.147 | 2.415 | -0.672 | 1.268 | < 0.0001 |
| Sphingomonadales-Sphingomonadaceae | 9.393 | -0.419 | -0.73 | -0.502 | 2.311 | -0.344 | -0.384 | < 0.0001 |
| **Spirochaetes** |  | | | | | | | |
| Spirochaetales-Spirochaetaceae | 35.346 | -3.93 | 1.358 | -3.405 | 2.216 | 2.732 | 0.761 | < 0.0001 |
| **Synergistetes** |  | | | | | | | |
| Synergistales-Synergistaceae | 29.861 | -3.382 | 1.365 | -2.287 | 3.228 | 1.981 | -1.524 | < 0.0001 |
| **Tenericutes** |  | | | | | | | |
| Unclassified Izimaplasmatales | 37.546 | -1.561 | -0.745 | -2.745 | 5.123 | -1.976 | 1.866 | < 0.0001 |
| Anaeroplasmatales-Anaeroplasmataceae | 27.695 | -1.946 | -1.022 | 0.1 | -2.723 | 2.173 | 3.793 | < 0.0001 |
| Unclassified Mollicutes RF39 | 18.939 | -0.153 | -0.807 | -2.732 | 2.209 | -0.971 | 2.998 | < 0.0001 |
| Unclassified T2WK15B57 | 16.73 | -0.701 | 3.654 | -1.118 | -0.854 | -0.967 | -0.601 | < 0.0001 |
| Mycoplasmatales-Mycoplasmataceae | 10.616 | -0.643 | -0.819 | -0.819 | -0.739 | 2.226 | 1.224 | < 0.0001 |
| **Verrucomicrobia** |  | | | | | | | |
| Verrucomicrobiales-Akkermansiaceae | 38.655 | 1.904 | -0.713 | 4.682 | -3.467 | -2.985 | 0.309 | < 0.0001 |
| Opitutales-Puniceicoccaceae | 33.275 | -1.165 | -1.676 | -2.048 | 5.159 | 1.24 | -1.449 | < 0.0001 |
| **Genus** | **Score** | **Fossa** | **Sportive lemur** | **Verreaux’s sifaka** | **Brown lemur** | **Bush pig** | **Zebu cattle** | ***P*** |
| *Ruminococcaceae UCG-014* | 54.694 | -3.996 | 5.54 | 2.384 | -1.474 | -1.567 | -3.1 | < 0.0001 |
| *Synergistes* | 54.018 | -2.708 | 5.643 | 2.205 | -0.794 | -3.302 | -3.18 | < 0.0001 |
| *Ruminiclostridium 9* | 52.307 | -2.823 | -4.266 | 3.058 | 5.092 | -0.129 | -1.675 | < 0.0001 |
| *Ruminococcus 1* | 50.696 | -2.751 | 5.643 | -4.526 | 1.568 | 0.407 | -1.157 | < 0.0001 |
| *Christensenellaceae R-7 group* | 49.745 | -3.571 | -2.795 | 1.817 | -2.632 | 4.157 | 3.21 | < 0.0001 |
| *Ruminococcaceae NK4A214 group* | 49.035 | -2.99 | 2.407 | -4.673 | -0.885 | 3.876 | 2.593 | < 0.0001 |
| *Candidatus_Stoquefichus* | 48.526 | -2.253 | -3.866 | 5.138 | 2.494 | 0.268 | -2.704 | < 0.0001 |
| *Alistipes* | 47.956 | -1.652 | 5.537 | -3.49 | -3.024 | 0.084 | 2.268 | < 0.0001 |
| *Lachnospiraceae NK3A20_group* | 47.711 | -2.157 | -3.452 | 5.585 | 2.311 | -2.041 | -1.345 | < 0.0001 |
| *Mailhella* | 47.467 | -2.075 | -3.309 | 3.709 | -2.919 | 0.269 | 4.389 | < 0.0001 |
| *Treponema 2* | 46.864 | -2.526 | -4.173 | -1.825 | 4.059 | 2.298 | 2.778 | < 0.0001 |
| *Prevotellaceae UCG-001* | 46.018 | -2.261 | -3.694 | 5.594 | 1.481 | -2.229 | 0.169 | < 0.0001 |
| *Phascolarctobacterium* | 45.899 | -3.93 | 3.169 | 4.245 | -0.986 | -2.022 | -2.624 | < 0.0001 |
| *Enterorhabdus* | 45.664 | -1.308 | 5.643 | -3.21 | 1.983 | -1.91 | -2.225 | < 0.0001 |
| *Akkermansia* | 44.296 | -1.621 | 5.557 | -2.666 | -2.095 | -2.028 | 2.243 | < 0.0001 |
| *Ruminococcaceae UCG-005* | 44.201 | -2.937 | -3.648 | -0.416 | -0.032 | 3.548 | 4.143 | < 0.0001 |
| *Collinsella* | 43.878 | 2.532 | -3.435 | 4.056 | 2.042 | -2.713 | -2.62 | < 0.0001 |
| *Desulfovibrio* | 43.442 | -2.284 | -3.701 | 4.761 | 1.596 | 1.516 | -2.639 | < 0.0001 |
| *Marvinbryantia* | 42.885 | -2.209 | 4.465 | -3.71 | 1.705 | 1.832 | -2.766 | < 0.0001 |
| *Campylobacter* | 42.317 | -1.716 | -2.901 | 5.012 | 2.6 | -2.064 | -2.014 | < 0.0001 |
| *Defluviitaleaceae UCG-011* | 42.296 | -1.602 | 5.643 | -2.491 | -2.033 | -1.94 | 1.742 | < 0.0001 |
| *Pyramidobacter* | 42.293 | -1.597 | -2.702 | 5.578 | 1.614 | -2.024 | -1.988 | < 0.0001 |
| *Prevotellaceae UCG-004* | 42.209 | -2.01 | -3.327 | 2.865 | 0.207 | -2.417 | 4.529 | < 0.0001 |
| *Clostridium sensu stricto 1* | 42.183 | 3.996 | -3.122 | -1.81 | -2.273 | 3.408 | 1.8 | < 0.0001 |
| *Lachnospiraceae FE2018 group* | 41.454 | -1.8 | 5.643 | 1.116 | -2.408 | -2.032 | -2.056 | < 0.0001 |
| *Prevotellaceae UCG-003* | 40.901 | -1.807 | -2.761 | -2.789 | 4.208 | 0.238 | 3.477 | < 0.0001 |
| *Family XIII UCG-001* | 40.811 | -2.422 | 4.195 | 3.013 | -1.165 | -2.734 | -2.782 | < 0.0001 |
| *Acetitomaculum* | 40.402 | -0.627 | -2.907 | 5.553 | -2.912 | 1.355 | -0.783 | < 0.0001 |
| *Parabacteroides* | 40.245 | -3.588 | 4.628 | -1.625 | 2.01 | -0.087 | -2.778 | < 0.0001 |
| *Anaerostipes* | 40.188 | -1.452 | -2.245 | -2.237 | 3.975 | 3.997 | -1.66 | < 0.0001 |
| *Oscillibacter* | 40.088 | -1.087 | -2.868 | -3.392 | 2.24 | 1.959 | 4.247 | < 0.0001 |
| *Anaeroplasma* | 40.045 | -1.336 | -2.373 | -2.246 | 4.561 | -1.56 | 3.299 | < 0.0001 |
| *Lachnospiraceae NK4A136 group* | 40.044 | 0.754 | -3.371 | -3.344 | 2.835 | 4.146 | 0.325 | < 0.0001 |
| *Alloprevotella* | 40.04 | -2.283 | -4.331 | 1.257 | 4.706 | -0.577 | 1.06 | < 0.0001 |
| *Candidatus*  *Methanogranum* | 39.593 | -2.02 | 3.44 | -3.375 | 3.854 | -0.291 | -2.398 | < 0.0001 |
| *Escherichia/Shigella* | 39.474 | 2.617 | -3.942 | 4.313 | -2.706 | 0.79 | -0.478 | < 0.0001 |
| *Hydrogenoanaerobacterium* | 38.142 | -1.896 | -2.962 | 3.617 | -2.55 | 3.685 | 0.076 | < 0.0001 |
| *Flavonifractor* | 38.12 | -1.536 | -2.083 | -2.172 | 3.106 | -1.412 | 4.529 | < 0.0001 |
| *Cerasicoccus* | 37.867 | -1.479 | -2.108 | 5.643 | -2.074 | -1.569 | 0.833 | < 0.0001 |
| *Lachnospiraceae UCG-001* | 37.733 | -1.461 | -2.172 | 5.632 | -1.873 | 0.837 | -1.725 | < 0.0001 |
| *Prevotella 7* | 37.527 | -1.368 | -2.298 | 5.643 | -1.836 | 0.76 | -1.628 | < 0.0001 |
| *Erysipelotrichaceae UCG-004* | 37.399 | -1.469 | -1.85 | -2.235 | 5.159 | -1.506 | 2.015 | < 0.0001 |
| *Rikenellaceae RC9 gut group* | 37.332 | -3.084 | 2.018 | -4.145 | 1.024 | 0.624 | 3.581 | < 0.0001 |
| *Solobacterium* | 37.225 | -1.154 | -2.009 | -2.855 | 4.993 | 2.255 | -0.864 | < 0.0001 |
| *Methanobrevibacter* | 37.023 | -1.206 | -1.925 | -1.931 | -1.601 | 4.322 | 3.369 | < 0.0001 |
| *Ruminococcaceae UCG-002* | 36.928 | -1.1 | -1.969 | -2.044 | -1.512 | 4.358 | 3.334 | < 0.0001 |
| *Family_XIII_AD3011_group* | 36.919 | -1.192 | -1.91 | -1.902 | -1.66 | 4.263 | 3.429 | < 0.0001 |
| *Prevotellaceae NK3B31 group* | 36.431 | -1.765 | 0.337 | -3.106 | 5.01 | 1.253 | -1.952 | < 0.0001 |
| *Fibrobacter* | 35.607 | -1.402 | -2.163 | 5.643 | -1.593 | -1.406 | 0.122 | < 0.0001 |
| *Paeniclostridium* | 35.495 | 3.996 | -2.451 | -1.953 | -2.03 | 1.34 | 2.934 | < 0.0001 |
| *Shuttleworthia* | 35.486 | -1.666 | -0.958 | 5.332 | -2.418 | 0.846 | -2.072 | < 0.0001 |
| *Denitrobacterium* | 35.144 | -2.735 | -4.452 | 3.186 | 0.896 | 1.959 | 1.011 | < 0.0001 |
| *Anaerobiospirillum* | 35.125 | -1.296 | -2.041 | 5.638 | -0.531 | -1.408 | -1.325 | < 0.0001 |
| *GCA-900066575* | 34.582 | -1.235 | -2.009 | -1.966 | 4.831 | -1.559 | 2.098 | < 0.0001 |
| *Acetatifactor* | 34.265 | -1.168 | -1.624 | 5.643 | -1.328 | -1.17 | -1.293 | < 0.0001 |
| *Anaerofilum* | 34.214 | -1.023 | -1.696 | 5.643 | -1.478 | -1.056 | -1.275 | < 0.0001 |
| *Senegalimassilia* | 34.201 | -0.968 | 5.643 | -1.699 | -1.381 | -1.303 | -1.183 | < 0.0001 |
| *Bacteroides* | 33.943 | 1.21 | 4.911 | -3.043 | 0.148 | -2.669 | -0.894 | < 0.0001 |
| *Romboutsia* | 33.4 | 3.181 | -2.441 | -2.038 | -2.002 | 1.794 | 3.26 | < 0.0001 |
| *Anaerovibrio* | 33.39 | -1.103 | -1.973 | -1.778 | 5.158 | 1.185 | -1.414 | < 0.0001 |
| *Tyzzerella_3* | 32.9 | -1.889 | -3.145 | 4.426 | 1.142 | -2.175 | 0.945 | < 0.0001 |
| *Blautia* | 32.618 | 3.044 | -2.496 | -2.523 | 2.317 | 2.692 | -1.764 | < 0.0001 |
| *Intestinimonas* | 31.884 | -1.073 | -1.937 | -2.033 | 4.788 | -1.281 | 1.719 | < 0.0001 |
| *Catenisphaera* | 31.858 | -0.617 | -1.643 | -2.319 | 4.823 | 1.606 | -1.619 | < 0.0001 |
| *Bilophila* | 31.755 | -1.23 | -1.995 | 5.203 | -1.678 | -1.442 | 0.443 | < 0.0001 |
| *Ruminococcaceae UCG-010* | 31.334 | -1.015 | -1.703 | -1.655 | -1.525 | 2.287 | 4.529 | < 0.0001 |
| *Ruminiclostridium* | 31.174 | -1.62 | 4.046 | -2.501 | -1.033 | -1.916 | 2.598 | < 0.0001 |
| *Sutterella* | 31.16 | 2.075 | -0.785 | -3.094 | 4.115 | 0.567 | -2.252 | < 0.0001 |
| *Ruminococcaceae UCG-009* | 31.025 | -0.943 | -1.697 | -1.66 | -1.527 | 2.224 | 4.529 | < 0.0001 |
| *Oribacterium* | 30.691 | -1.148 | -2.058 | -0.818 | 5.159 | 0.147 | -1.426 | < 0.0001 |
| *Lachnoclostridium* | 30.659 | 1.995 | -1.958 | -3.811 | 3.779 | 0.156 | 0.966 | < 0.0001 |
| *Megasphaera* | 29.898 | -1.118 | -1.743 | -1.669 | 4.9 | 1.033 | -1.376 | < 0.0001 |
| *Lachnospiraceae UCG-006* | 29.518 | -0.842 | -1.216 | -1.41 | 5.159 | -0.966 | -0.896 | < 0.0001 |
| *Ruminococcaceae UCG-004* | 29.452 | -1.18 | -1.661 | -1.653 | -1.353 | 2.643 | 4.068 | < 0.0001 |
| *Olsenella* | 29.447 | -0.991 | -1.315 | -1.182 | 5.159 | -1.033 | -0.858 | < 0.0001 |
| *Ruminococcaceae UCG-013* | 29.049 | -1.214 | -2.118 | -1.927 | 0.003 | 1.645 | 4.463 | < 0.0001 |
| *Megamonas* | 29.048 | -0.701 | -1.285 | -1.374 | 5.159 | -0.901 | -1.043 | < 0.0001 |
| *p-1088-a5_gut_group* | 28.472 | -1.047 | -1.569 | -1.657 | -1.52 | 3.811 | 2.862 | < 0.0001 |
| *Erysipelatoclostridium* | 28.095 | 2.932 | 1.831 | -2.456 | -2.242 | -1.729 | 2.474 | < 0.0001 |
| *Terrisporobacter* | 27.708 | -0.253 | -1.748 | -1.788 | -1.603 | 3.965 | 2.49 | < 0.0001 |
| *Mogibacterium* | 27.431 | 3.996 | -1.354 | -1.521 | -1.217 | -1.091 | 2.534 | < 0.0001 |
| *Coprococcus_3* | 27.425 | 2.365 | -1.914 | -1.956 | -1.701 | 0.975 | 3.696 | < 0.0001 |
| *Odoribacter* | 27.278 | -1.271 | 3.784 | -2.06 | -1.646 | -1.565 | 2.414 | < 0.0001 |
| *Lachnospiraceae_UCG-010* | 27.189 | -1.698 | -2.814 | 4.022 | 0.627 | 1.206 | -1.926 | < 0.0001 |
| *Roseburia* | 26.99 | -2.022 | -0.222 | 4.53 | -2.678 | -0.822 | 0.291 | < 0.0001 |
| *Candidatus_Soleaferrea* | 26.363 | -0.898 | -1.485 | -1.557 | -1.444 | 2.143 | 4.083 | < 0.0001 |
| *Breznakia* | 26.226 | -1.006 | -1.762 | -1.707 | 4.168 | -1.331 | 1.794 | < 0.0001 |
| *Negativibacillus* | 26.047 | 2.472 | -1.316 | -1.493 | -1.346 | -0.995 | 3.846 | < 0.0001 |
| *Butyricimonas* | 26.035 | -0.429 | 4.795 | -1.752 | -1.509 | -0.4 | -1.295 | < 0.0001 |
| *Sphaerochaeta* | 26.009 | -0.818 | -1.285 | -1.198 | 4.858 | -0.803 | -0.915 | < 0.0001 |
| *Cloacibacillus* | 25.958 | -0.972 | -1.392 | 4.839 | -1.246 | -0.981 | -1.036 | < 0.0001 |
| *Faecalibacterium* | 25.767 | -1.052 | -1.722 | -1.64 | 4.33 | 1.384 | -1.199 | < 0.0001 |
| *Lachnospiraceae UCG-002* | 25.477 | -0.439 | -0.013 | -1.76 | 4.665 | -1.39 | -1.326 | < 0.0001 |
| *Caproiciproducens* | 25.436 | -2.108 | -0.998 | 3.921 | -2.898 | 1.014 | 0.505 | < 0.0001 |
| *Lysinibacillus* | 25.252 | 1.315 | -1.777 | -1.81 | -1.527 | 0.98 | 4.067 | < 0.0001 |
| *Lachnospiraceae UCG-008* | 24.672 | -0.984 | -1.628 | -1.651 | 4.285 | 1.164 | -1.09 | < 0.0001 |
| *Mycoplasma* | 24.477 | -0.796 | -1.103 | -1.302 | 4.638 | -0.783 | -0.801 | < 0.0001 |
| *Subdoligranulum* | 24.144 | -0.862 | -1.158 | -1.086 | 4.591 | -0.791 | -0.883 | < 0.0001 |
| *Cellulosilyticum* | 23.982 | -0.608 | -1.016 | -1.179 | -1.009 | 4.529 | -0.109 | < 0.0001 |
| *Tyzzerella* | 23.834 | -0.671 | -0.843 | -0.903 | -0.76 | -0.897 | 4.529 | < 0.0001 |
| *Tyzzerella_4* | 23.765 | -0.423 | -1.05 | -0.967 | -0.966 | -0.544 | 4.529 | < 0.0001 |
| *dgA-11_gut_group* | 23.663 | -0.619 | -0.958 | -0.96 | -0.838 | -0.639 | 4.529 | < 0.0001 |
| *Dorea* | 23.614 | -0.695 | -0.946 | -0.897 | -0.754 | -0.761 | 4.529 | < 0.0001 |
| *Solibacillus* | 23.557 | -1.048 | -1.065 | -1.761 | -1.358 | 3.323 | 2.669 | < 0.0001 |
| *Clostridioides* | 23.483 | -0.531 | -0.918 | -0.921 | -0.945 | -0.693 | 4.529 | < 0.0001 |
| *Agathobacter* | 23.413 | -0.6 | -0.864 | -1.012 | -0.792 | -0.763 | 4.529 | < 0.0001 |
| *Oxalobacter* | 23.316 | -1.775 | 1.172 | 3.759 | -0.497 | -2.068 | -1.942 | < 0.0001 |
| *Pygmaiobacter* | 23.27 | -0.6 | -1.005 | -0.997 | -0.83 | -0.559 | 4.528 | < 0.0001 |
| *Kurthia* | 23.225 | -0.546 | -0.987 | -1.164 | -0.985 | -0.243 | 4.525 | < 0.0001 |
| *Methanocorpusculum* | 22.348 | -0.816 | -1.388 | -1.277 | -1.022 | 1.079 | 4.125 | < 0.0001 |
| *Ruminiclostridium_5* | 21.869 | -1.508 | 1.856 | -2.201 | -1.947 | 0.841 | 3.138 | < 0.0001 |
| *Anaerofustis* | 21.458 | -1.434 | -2.382 | 3.57 | 0.807 | -1.796 | 0.662 | < 0.0001 |
| *Ruminococcus_2* | 21.447 | -0.802 | -1.354 | -1.325 | -1.1 | 3.818 | 1.481 | < 0.0001 |
| *Parasutterella* | 20.899 | 0.544 | 1.977 | -2.158 | -1.696 | -1.393 | 3.07 | < 0.0001 |
| *Paraprevotella* | 20.716 | -1.161 | 2.267 | -1.888 | 2.947 | -1.427 | -1.377 | < 0.0001 |
| *Anaerovorax* | 20.006 | -0.635 | -1.111 | -1.281 | -0.913 | 0.706 | 3.875 | < 0.0001 |
| *Oscillospira* | 19.908 | -0.803 | -1.098 | -1.342 | 0.651 | 3.903 | -0.897 | < 0.0001 |
| *Peptostreptococcus* | 19.661 | 3.996 | -0.77 | -0.725 | -0.581 | -0.566 | -0.435 | < 0.0001 |
| *Butyricicoccus* | 19.603 | -0.374 | -0.338 | -2.19 | 3.656 | -1.627 | 0.942 | < 0.0001 |
| *GCA-900066225* | 19.446 | -0.813 | -1.104 | -1.122 | -1.084 | 3.781 | 0.945 | < 0.0001 |
| *Intestinibacter* | 19.381 | -0.64 | -0.89 | -0.806 | -0.649 | 4.024 | -0.607 | < 0.0001 |
| *Actinomyces* | 19.349 | 3.996 | -0.847 | -0.742 | -0.607 | -0.296 | -0.558 | < 0.0001 |
| *Peptoclostridium* | 19.289 | 3.996 | -0.674 | -0.74 | -0.681 | -0.542 | -0.446 | < 0.0001 |
| *Lachnospiraceae NC2004 group* | 18.891 | -0.359 | -0.936 | -0.867 | -0.71 | 4.001 | -0.629 | < 0.0001 |
| *Clostridium sensu stricto 2* | 18.774 | -0.389 | -0.665 | -0.954 | -0.857 | 3.94 | -0.604 | < 0.0001 |
| *Lachnospira* | 18.588 | -0.547 | -0.782 | -0.912 | -0.642 | 3.935 | -0.611 | < 0.0001 |
| *Ruminobacter* | 18.062 | 0.147 | -0.908 | -1.057 | -0.846 | -0.535 | 3.817 | < 0.0001 |
| *Streptococcus* | 17.816 | 2.552 | -3.509 | 1.347 | 0.052 | 0.631 | -0.248 | < 0.0001 |
| *Turicibacter* | 17.797 | 0.08 | -1.096 | -1.393 | -1.048 | 0.565 | 3.659 | < 0.0001 |
| *Saccharofermentans* | 17.255 | -0.686 | -0.774 | -0.853 | -0.677 | -0.354 | 3.756 | < 0.0001 |
| *Ruminococcaceae UCG-011* | 15.828 | -0.394 | -0.749 | -0.807 | -0.602 | -0.537 | 3.51 | < 0.0001 |
| *Clostridium_sensu_stricto_3* | 15.642 | -0.431 | -0.899 | -0.604 | -0.559 | 3.45 | -0.561 | < 0.0001 |
| *Ruminiclostridium 6* | 15.48 | -0.328 | -0.993 | -0.93 | -0.62 | 3.386 | 0.004 | < 0.0001 |
| *Erysipelotrichaceae UCG-003* | 15.419 | -0.396 | -0.734 | -0.643 | -0.781 | 3.442 | -0.487 | < 0.0001 |
| *Peptoniphilus* | 15.05 | 3.348 | -0.729 | -0.699 | -0.518 | -0.282 | -0.302 | < 0.0001 |
| *Fusobacterium* | 14.66 | 3.332 | -0.728 | -0.541 | -0.52 | -0.422 | -0.345 | < 0.0001 |
| *Harryflintia* | 14.111 | -0.427 | -0.626 | -0.73 | -0.652 | -0.509 | 3.317 | < 0.0001 |
| *Succinivibrio* | 14.072 | -0.36 | -0.63 | -0.853 | -0.584 | 3.286 | -0.455 | < 0.0001 |
| *Hathewaya* | 13.903 | 3.289 | -0.792 | -0.893 | -0.196 | -0.053 | -0.526 | < 0.0001 |
| *FD2005* | 13.535 | -0.645 | -0.887 | -0.728 | 3.214 | -0.665 | -0.414 | < 0.0001 |
| *Elusimicrobium* | 13.337 | -0.844 | -1.468 | -1.484 | 0.439 | 1.766 | 2.157 | < 0.0001 |
| *Helicobacter* | 13.151 | 2.497 | -1.624 | 0.789 | 0.825 | -0.976 | -1.127 | < 0.0001 |
| *DNF00809* | 12.902 | -0.489 | -1.173 | -0.916 | -0.735 | 2.682 | 1.189 | < 0.0001 |
| *Atopobium* | 12.204 | -0.772 | -1.149 | 2.646 | 0.777 | -1.108 | -0.96 | < 0.0001 |
| *Coprococcus_1* | 12.032 | -0.566 | -0.568 | -0.499 | -0.639 | 2.889 | -0.333 | < 0.0001 |
| *Lachnospiraceae FCS020_group* | 11.856 | -0.256 | -0.754 | -0.556 | -0.441 | -0.495 | 2.857 | < 0.0001 |
| *Lachnoclostridium_10* | 11.819 | -0.341 | -0.899 | -0.919 | -0.69 | 0.627 | 2.724 | < 0.0001 |
| *Erysipelotrichaceae UCG-002* | 11.694 | -0.323 | -0.604 | -0.587 | -0.659 | 2.848 | -0.328 | < 0.0001 |
| *Lactobacillus* | 11.638 | 1.839 | -0.813 | -0.931 | -0.779 | 2.012 | -0.575 | < 0.0001 |
| *Parvibacter* | 11.581 | -0.406 | -0.59 | -0.695 | -0.486 | -0.375 | 2.888 | < 0.0001 |
| *Slackia* | 11.477 | 2.296 | -1.786 | -0.097 | 0.386 | 1.073 | -1.191 | < 0.0001 |
| *Enterococcus* | 11.224 | 2.32 | 0.002 | -0.241 | -1.102 | 1.212 | -1.704 | 0.00014 |
| *Papillibacter* | 10.985 | -0.365 | 0.625 | -0.885 | -0.9 | -0.763 | 2.434 | 0.00014 |
| *Faecalitalea* | 10.8 | 2.617 | -0.391 | -0.632 | -0.397 | -0.251 | -0.332 | 0.00014 |
| *Actinobacillus* | 10.545 | -0.525 | -0.17 | -0.854 | 2.518 | -0.666 | -0.463 | 0.00014 |
| *Sarcina* | 9.956 | 2.496 | -0.613 | -0.625 | -0.358 | 0.224 | -0.475 | 0.00028 |
| *Acetobacter* | 9.493 | -0.375 | -0.566 | -0.722 | 2.429 | -0.493 | -0.338 | 0.0004 |
| *Lachnospiraceae AC2044_group* | 9.399 | -0.455 | -0.435 | -0.26 | -0.423 | 2.266 | -0.517 | 0.0004 |
| *Angelakisella* | 9.35 | -0.45 | -0.475 | -0.394 | -0.387 | 2.287 | -0.366 | 0.0004 |
| *Candidatus Methanomethylophilus* | 9.207 | -0.207 | -0.565 | -0.643 | -0.375 | 2.335 | -0.221 | 0.0004 |
| *Ruminococcaceae V9D2013_group* | 9.166 | -0.589 | 2.188 | -0.961 | 0.435 | -0.671 | -0.834 | 0.0004 |
| *Avibacterium* | 8.862 | -0.335 | -0.613 | -0.653 | 2.297 | -0.344 | -0.4 | 0.00053 |
| *Z20* | 8.286 | -0.305 | -0.406 | -0.477 | -0.445 | 2.202 | -0.327 | 0.0008 |
| *Acinetobacter* | 7.811 | -0.699 | 0.111 | 0.143 | -0.798 | 1.873 | -0.664 | 0.00118 |
| *Frateuria* | 7.754 | -0.187 | -0.351 | -0.572 | 1.833 | -0.507 | -0.266 | 0.00118 |
| *Bifidobacterium* | 7.587 | 1.924 | -0.706 | -0.612 | 0.303 | 0.005 | -0.404 | 0.00142 |
| *Clostridium_sensu_stricto_6* | 7.569 | -0.397 | -0.586 | -0.854 | -0.309 | 1.59 | 0.906 | 0.00142 |
| *Staphylococcus* | 7.537 | -0.206 | -0.151 | -1.64 | -0.247 | 1.506 | 1.188 | 0.00142 |
| *Barnesiella* | 7.355 | -0.393 | 1.85 | -0.553 | -0.412 | -0.419 | -0.382 | 0.00165 |
| *Corynebacterium_1* | 7.342 | 1.805 | -0.369 | -0.313 | -0.298 | -0.207 | -0.196 | 0.00165 |
| *Pseudomonas* | 7.1 | 1.273 | 0.993 | 0.419 | -0.551 | -1.607 | -0.647 | 0.0019 |
| *horsej-a03* | 7.025 | -0.093 | -0.3 | -0.29 | -0.454 | 1.672 | -0.338 | 0.00212 |
| *Thiopseudomonas* | 6.981 | -0.195 | -0.581 | -0.209 | -0.237 | 1.722 | -0.295 | 0.00212 |
| *Chishuiella* | 6.775 | -0.151 | -0.354 | -0.412 | -0.139 | 1.622 | -0.376 | 0.00247 |
| *Phoenicibacter* | 6.756 | -0.306 | 1.629 | -0.513 | -0.324 | -0.378 | -0.373 | 0.00247 |
| *Flavobacterium* | 6.574 | -0.223 | -0.3 | -0.415 | -0.417 | 1.719 | -0.156 | 0.00247 |
| *Bacillus* | 6.404 | -0.392 | -0.57 | 0.404 | -0.458 | 1.429 | -0.354 | 0.00247 |
| *Coprococcus_2* | 6.314 | -0.295 | -0.482 | -0.146 | -0.235 | -0.199 | 1.51 | 0.00392 |
| *Dubosiella* | 6.129 | 1.228 | -0.306 | -0.549 | -0.317 | 0.553 | -0.209 | 0.00392 |
| *Proteus* | 6.08 | 1.136 | -0.25 | -0.226 | -0.315 | 0.416 | -0.466 | 0.00392 |
| *Methylobacterium* | 6.075 | 1.118 | -0.281 | 0.843 | -0.454 | -0.686 | -0.483 | 0.00392 |
| *Pluralibacter* | 6.044 | -0.406 | -0.411 | -0.208 | 1.23 | -0.286 | 0.015 | 0.00392 |
| *Streptomyces* | 5.63 | 0.54 | -0.229 | -0.381 | -0.421 | 0.96 | -0.197 | 0.00543 |

**Table S3. Results of indicator species analysis showing the association between gut bacteria genera and host substrate preference (terrestriality versus arboreality).** Results pertain to genera having indicator values > 0.5 and significance at *P* < 0.05.

| **Indicator genera** | **Terrestriality Indicator value** | **Arboreality Indicator value** | ***P*** |
| --- | --- | --- | --- |
| *Lachnoclostridium* | 0.932 | - | 0.0001 |
| *Lachnospiraceae NK4A136 group* | 0.921 | - | 0.0001 |
| *Oscillibacter* | 0.904 | - | 0.0001 |
| *Rikenellaceae RC9 gut group* | 0.904 | - | 0.0012 |
| *Treponema 2* | 0.903 | - | 0.0001 |
| *Prevotellaceae UCG-003* | 0.853 | - | 0.0001 |
| *Alloprevotella* | 0.845 | - | 0.0004 |
| *Clostridium sensu stricto 1* | 0.824 | - | 0.0001 |
| *Solobacterium* | 0.816 | - | 0.0001 |
| *Ruminiclostridium 9* | 0.809 | - | 0.0201 |
| *Blautia* | 0.798 | - | 0.0005 |
| *Anaeroplasma* | 0.759 | - | 0.0001 |
| *Anaerostipes* | 0.759 | - | 0.0001 |
| *Paeniclostridium* | 0.759 | - | 0.0006 |
| *Flavonifractor* | 0.739 | - | 0.0002 |
| *GCA-900066575* | 0.718 | - | 0.0002 |
| *Faecalibacterium* | 0.651 |  | 0.0005 |
| *Mogibacterium* | 0.603 |  | 0.0342 |
| *Synergistes* | - | 0.980 | 0.0001 |
| *Family XIII UCG-001* | - | 0.927 | 0.0001 |
| *Lachnospiraceae FE2018 group* | - | 0.926 | 0.0001 |
| *Shuttleworthia* | - | 0.759 | 0.0002 |
| *Oxalobacter* | - | 0.733 | 0.0001 |
| *Acetatifactor* | - | 0.707 | 0.0001 |
| *Anaerofilum* | - | 0.707 | 0.0001 |
| *Senegalimassilia* | - | 0.707 | 0.0002 |
| *Anaerobiospirillum* | - | 0.702 | 0.0004 |
| *Prevotella 7* | - | 0.701 | 0.0009 |
| *Cerasicoccus* | - | 0.700 | 0.0005 |
| *Fibrobacter* | - | 0.688 | 0.0006 |
| *Bilophila* | - | 0.665 | 0.0009 |
| *Cloacibacillus* | - | 0.655 | 0.0003 |
| *Butyricimonas* | - | 0.638 | 0.0018 |
